# Utility of targeted deep sequencing for detecting circulating tumor DNA in pancreatic cancer patients

**DOI:** 10.1038/s41598-018-30100-w

**Published:** 2018-08-02

**Authors:** Gahee Park, Joo Kyung Park, Dae-Soon Son, Seung-Ho Shin, Yeon Jeong Kim, Hyo-Jeong Jeon, Jae Lee, Woong-Yang Park, Kwang Hyuck Lee, Donghyun Park

**Affiliations:** 1 Samsung Genome Institute, Samsung Medical Center, Seoul, 06351 Korea; 2 Department of Biomedical Sciences, Seoul National University College of Medicine, Seoul, 03080 Korea; 3 Department of Medicine, Samsung Medical Center, Sungkyunkwan University School of Medicine, Seoul, 06351 Korea; 4 Department of Health Sciences and Technology, Samsung Advanced Institute for Health Sciences & Technology, Sungkyunkwan University, Seoul, 06351 Korea; 5 Department of Molecular Cell Biology, Sungkyunkwan University School of Medicine, Suwon, 16419 Korea

## Abstract

Targeted deep sequencing across broad genomic regions has been used to detect circulating tumor DNA (ctDNA) in pancreatic ductal adenocarcinoma (PDAC) patients. However, since most PDACs harbor a mutation in KRAS, sequencing of broad regions needs to be systemically compared to analyzing only KRAS mutations for PDAC. Using capture-based targeted deep sequencing, we detected somatic tumor mutations in 17 fine needle aspiration biopsy and 69 longitudinal cell-free DNA (cfDNA) samples from 17 PDAC patients. KRAS mutations were detected in 10 out of 17 pretreatment patient plasma samples. Next, interrogation of genetic alterations in matched primary tumor samples detected ctDNA in 12 of 17 pretreatment plasma samples and cfDNA sequencing across the 83 target genes identified ctDNA in 15 of 17 cases (88.2% sensitivity). This improved sensitivity of ctDNA detection resulted in enhanced tumor burden monitoring when we analyzed longitudinal plasma samples. We found that cfDNA sequencing detected the lowest mutant allelic fractions and number of variants when complete response or partial response to chemotherapy was achieved. We demonstrated that ctDNA levels measured by targeted deep sequencing sensitively indicate the presence of cancer and correlate well with clinical responses to therapy and disease progression in PDAC patients.

## Introduction

Pancreatic ductal adenocarcinoma (PDAC) is one of the leading mortality-causing diseases internationally, which is due, at least in part, to the lack of a noninvasive biomarker for sensitive and specific disease detection^1^. Because a potentially curative operation that facilitates long-term survival is primarily successful for patients with the clinically localized disease^2,3^, numerous studies have attempted to identify a highly accurate blood-based biomarker for early detection of PDAC. Nonetheless, cancer antigen (CA) 19-9 remains the standard biomarker^4^, despite its unsatisfactory sensitivity and specificity for early detection of disease^5^, which also limits its role monitoring disease burden. Recently, circulating tumor DNA (ctDNA) has been proposed as an alternative to traditional noninvasive protein biomarkers due to its potential for use in a wide range of clinical applications for various cancers, including PDAC^6–8^. Because ctDNA is released from tumor cells into the blood, the presence of ctDNA, as detected through mutations harbored by the original tumor, is indicative of a tumor and relative tumor burden^7,9^. Previous studies have reported highly sensitive and specific genetic profiling of plasma DNA, suggesting that the use of ctDNA as a liquid biopsy might significantly improve current systems of tumor diagnosis^10^, tumor progression monitoring^9^, targeted therapies^11^, and early-stage detection^12^. While expected to revolutionize cancer diagnoses in general^13^, liquid biopsy based on ctDNA analysis is even more anticipated for particular cancer types, such as PDAC, where biopsy is risky and often untenable.

Among the various methods of detecting ctDNA, digital PCR methods, including BEAMing (beads, emulsion, amplification, and magnetics), have been effectively utilized to detect a limited number of specific target variants, including KRAS, EGFR, and PIK3CA hotspot mutations, across various cancers^7,11,14–18^. Because mutations in KRAS are observed in >90% of PDAC^1^ and are likely to be clonal mutations present in the majority of cancer cells, they are often identified in plasma as a ctDNA benchmark for PDAC^6,18–22^. This unique mutational feature of PDAC renders digital PCR very attractive for ctDNA detection in PDAC patients via interrogating a few KRAS hotspots^6,8,18,20^.

Despite the considerably high sensitivity of digital PCR, the detection of KRAS mutations in plasma using this method has often fallen short of high expectations, as the ctDNA detection rate has averaged as low as 50%^6,19–21,23^. This limitation may be a result of there being low allelic fractions of KRAS mutations in a subset of PDACs^24^. In fact, the allelic fractions of KRAS mutations in PDAC biopsy samples range from homozygous wild-type to ≥100% mutated KRAS, indicating KRAS-mutated populations might be subclonal in a significant fraction of PDAC patients^1,24^.

Targeted deep sequencing has been employed to interrogate tumor variants across relatively broad genomic regions that include many cancer-associated target genes using blood samples from patients with various types of cancer^9,10,22,25,26^. It is now beyond doubt that ctDNA sequencing analysis of broad genomic regions facilitates evaluations of the tumor burden^25,27^, intra-tumor genetic heterogeneity^28^, emergence of resistant mutations^29,30^, and clonal expansion^31^ during disease progression. Conversely, interrogation of broad genomic regions requires more resources for generation of raw data and subsequent downstream analysis. Additionally, it might generate more frequent false positives^32^, unless detection sensitivity is compromised to a certain degree. Therefore, deep sequencing dozens to hundreds of cancer-related genes have to be carefully evaluated to determine if the benefits outweigh the disadvantages, especially for PDAC, where at least one of a few KRAS variants are observed in most cases. Here, we evaluated the benefits of investigating 83 target genes to detect ctDNA in pancreatic cancer patients and compared the method to testing either KRAS hotspots or genetic variants in their matched biopsy samples.

## Results

### Generation of targeted deep sequencing data for PDAC patients

To evaluate ctDNA detection by targeted deep sequencing and its clinical utilities, 17 PDAC patients with available tumor biopsy samples underwent blood draws for cell-free DNA (cfDNA) testing (Table 1). We profiled a total of 120 samples from these 17 patients consisting of 17 fine needle aspiration (FNA) biopsies, 34 peripheral blood leucocyte (PBL) samples, and 69 plasma samples (Supplementary Fig. S1 and Table S1). Of this sequencing data, 17 plasma and 17 PBL samples obtained prior to treatment were recently published in our study analyzing sequencing background noise^33^.

**Table 1:** Summary of clinical data for the 17 pancreatic cancer patients enrolled in this study.

ID	Age	Sex	Histology	Stage	TNM	Metastatic Site(s)	OS	Death	Regimen	Treatment Modality
P2	55	F	PDAC	IIB	T3N1M0	ND	7.9	Yes	GEM	Op & Chemotherapy
P5	59	F	PDAC	III	T4N1M0	ND	15.4	Yes	1st: FOLFIRINOX 2nd: FOLFIRINOX/GEM-P	Chemotherapy
P7	59	F	PDAC	III	T4N1M0	ND	12.5	Yes	GEM-E	Chemotherapy
P10	59	M	PDAC	IV	T3N1M1	Liver	2.3	Yes	—	Best supportive care only
P11	48	M	PDAC	IIB	T3N1M0	ND	20.2	Yes	1st: CCRT-5FU 2nd: 5FU-LV 3rd: GEM-Abraxane	Op & Chemotherapy
P21	59	M	PDAC	III	T4N1M0	ND	11.9	Yes	FOLFIRINOX	Chemotherapy
P23	59	F	PDAC	III	T4N1M0	ND	21.2	No	GEM-E	Chemotherapy
P27	59	F	PDAC	III	T4N1M0	ND	21.2	No	GEM-E	Chemotherapy
P28	75	M	PDAC	III	T4N1M0	ND	8.9	Yes	GEM	Chemotherapy
P29	59	M	PDAC	IV	T4N0M1	Liver/ adrenal mets	7.4	Yes	1st: GEM-E 2nd: XELOX	Chemotherapy
P31	60	M	PDAC	III	T4N0M0	ND	18.1	No	GEM-E	Chemotherapy
P32	59	F	PDAC	IV	T3N1M1	Liver	16.7	Yes	—	Best supportive care only
P36	60	M	PDAC	IV	T3N1M1	Lung mets	9.4	Yes	GEM	Chemotherapy
P37	59	F	PDAC	IIB	T2N1M0	ND	8.2	Yes	TS-1	Chemotherapy
P42	59	M	PDAC	IV	T4N1M1	Liver	14.5	Yes	1st: FOLFIRINOX 2nd: GEM-E 3rd: TS-1	Chemotherapy
P43	59	F	PDAC	IV	T4N1M1	Peritoneal seeding/ ascites	17.7	Yes	FOLFIRINOX	Chemotherapy
P46	60	M	PDAC	III	T4N1M0	ND	17.6	No	—	Best supportive care only

CCRT, concurrent chemoradiation therapy; E, erlotinib; FU, fluorouracil; FOLFIRINOX, fluorouracil, leucovorin, oxaliplatin, and irinotecan; GEM, gemcitabine; mets, metastasis; ND, not detected; Op, operative procedure; P, cisplatin; PDAC, pancreatic adenocarcinoma; TNM, tumor/node/metastasis; TS-1, titanium silicate-1; XELOX, capecitabine plus oxaliplatin.

To achieve a mean sequencing depth of ~10,000X (prior to duplicate removal) in a cost-effective manner, we designed a pool of RNA baits targeting 83 cancer-associated genes, including hotspots for pancreatic cancer (Supplementary Table S2). DNA libraries were constructed from plasma DNA and matched germline DNA (i.e., PBL genomic DNA) samples and sequenced on Illumina HiSeq. 2500 as we previously reported^33^. After excluding PCR duplication, the unique coverage depths for the biopsy, plasma DNA, and germline DNA samples averaged 987.15X (790.32–1476.55X), 2227.25X (490.27–5532.04X), and 1928.55X (1042.12–2843.48X), respectively (Supplementary Table S1).

From these data sets, we detected mutations in FNA biopsies (M_FNA_) and plasma samples (M_P_) as described below. Hereafter, depending on the method of detecting M_P_, the following notations will be used: M_P/KRAS_, mutations detected in KRAS; M_P/FNA_, mutations detected in M_FNA_; M_P/TR-BF_, mutations detected in target regions in a biopsy-free manner; M_P/TR_, mutations detected in target regions combined with M_P/FNA_ and M_P/TR-BF_.

### KRAS mutations

**Figure 1:**
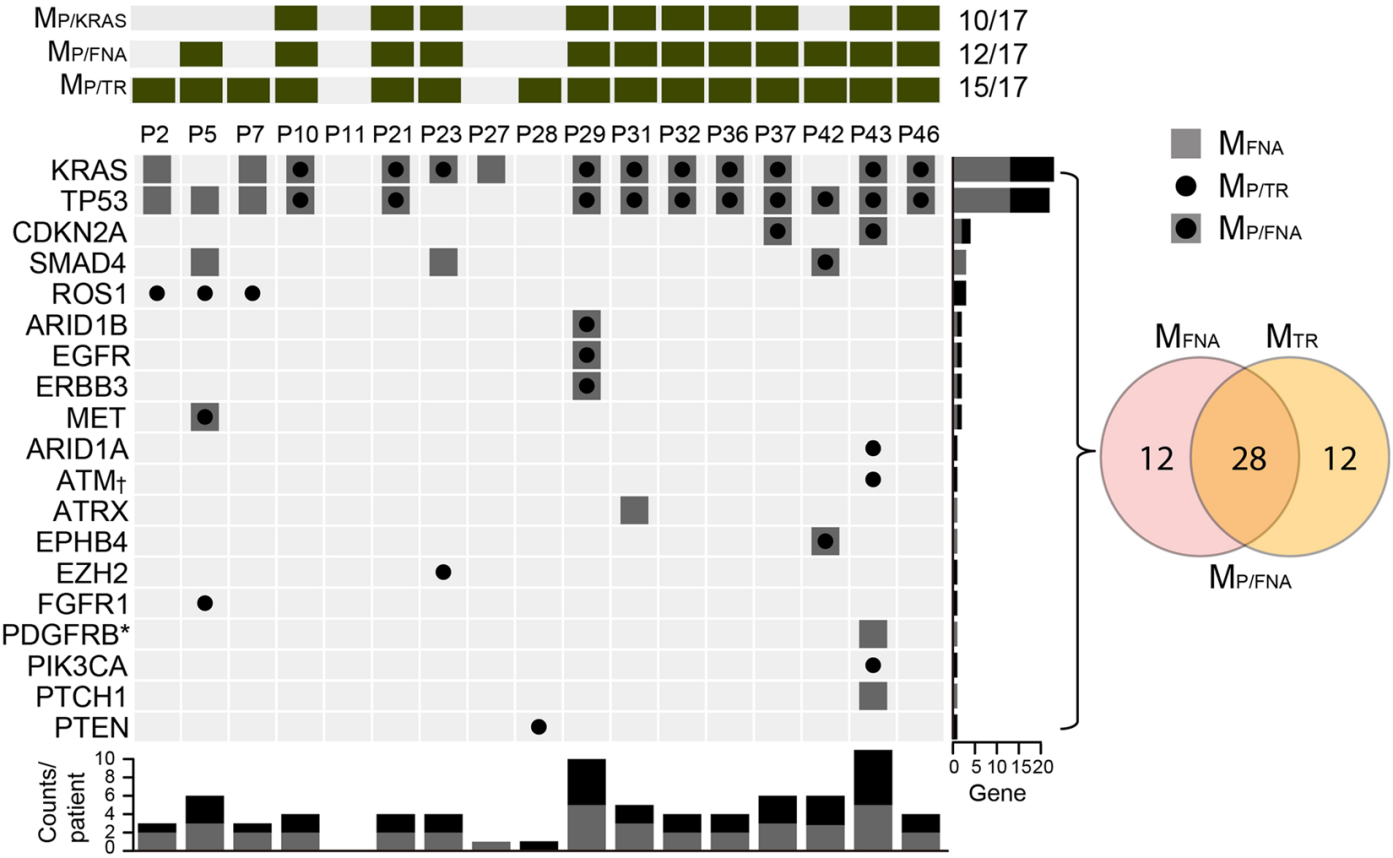
Detection of ctDNA in pretreatment plasma samples from 17 pancreatic cancer patients. The top panel summarizes the mutations detected in the 17 patients based on method (i.e., M_P/KRAS_, M_P/FNA_, and M_P/TR_). While interrogation of KRAS hotspots (M_P/KRAS_) detected ctDNA from 10 patients, analyses of variants detected from FNA samples (M_P/FNA_) and broad genomic target regions (M_P/TR_) detected tumor variants in 12 and 15 plasma samples, respectively. The oncoprint chart presents the M_FNA_ and M_P/TR_ at the gene level. If a variant was detected in both the M_FNA_ and M_P/TR_, the variant also corresponded with M_P/FNA_. The number of affected genes for each patient was plotted at the bottom of the chart. The number of samples that harbored a mutation in each gene is plotted on the right side of the chart. Two * and four † independent mutations in PDGFRB and ATM were detected in the P43 FNA and plasma samples, respectively.

First, because most PDACs carry a KRAS mutation, we determined whether these mutations were present in 17 pretreatment patient samples. We detected KRAS mutations in 10 plasma (58.8%) and 13 FNA samples (76.5%) (Fig. 1). Because the allelic fractions of somatic variants are usually notably lower in plasma samples than in tissue samples, the lower detection rate of KRAS mutations in plasma samples than in FNA samples was the expected result. The mean ± standard error of the mean (SEM) of KRAS mutant allele frequencies (MAFs) were 21.18% ± 4.06 in FNA samples and 2.02% ± 0.67 in plasma samples.

To exclude the possibility that the moderate M_P/KRAS_ (plasma DNA mutations in KRAS) detection rate was due to poor analytic sensitivity of the method, we evaluated the analytic detection sensitivity and specificity of the targeted deep sequencing method for KRAS mutations using 62 consecutive samples from 14 patients. Based on droplet digital PCR (ddPCR) analysis of the samples, the targeted deep sequencing method had a 95.7% sensitivity with a 95% confidence interval (CI) of 78.1 to 99.9% and a 100% specificity with a 95% CI of 91.2 to 100% for detection of KRAS mutations (Supplementary Table S3 and S4), thus negating the possibility of poor sensitivity.

Therefore, evaluating only M_P/KRAS_ may be limited by the moderate sensitivity of the method for detecting ctDNA, despite the majority of PDAC patients carrying KRAS mutations.

### Detection of single nucleotide variants (SNVs) across broad genomic target regions

Utilizing customized capture-based targeted sequencing, we detected a total of 40 mutations in FNA samples (M_FNA_) from 17 patients, including 2 specimens from patients P11 and P28 who had no significant mutations (Supplementary Table S5). In order to detect ctDNA, the 40 M_FNA_ were statistically evaluated to determine the significance of these mutations in the matched pretreatment plasma DNA samples (p < 0.001). Among the 40 mutations found in the FNA samples, 28 were detected in the corresponding plasma samples (M_P/FNA_) and were significantly above the background noise of the plasma DNA, resulting in a 70.0% detection sensitivity with an average MAF of 1.60% ± 0.31 (mean ± SEM) (Fig. 1a and Supplementary Table S6).

Next, we attempted to detect ctDNA by analyzing broad genomic target regions in a biopsy-free manner (M_P/TR-BF_) as described in previous studies with a minor modification (Materials and Methods)^9,34^. This approach aimed to detect somatic variants without profiling tissue samples, eschewing the necessity of biopsy. From this analysis, we detected 27 M_P/TR-BF_ in pretreatment plasma samples, including 15 mutations concordantly detected in FNA samples (Fig. 1b and Supplementary Table S6) with a median MAF of 3.54% ± 1.38. Twelve mutations were detected in the plasma DNA samples, but not in the matched FNA biopsy samples.

From the M_P/TR-BF_ detected in the plasma samples either prior to treatment or peri/post-treatment, but not in their matched FNAs, we selected two variants (ROS1 p.I1967V and RB1 p.251X) from two patients (P2 and P5) and performed ddPCR to validate their presence in 8 plasma DNA samples (Supplementary Table S3). Consistent with the cfDNA sequencing results, these mutations were detected in consecutive plasma samples from these patients, indicating that variants detected only in plasma were unlikely to be false positives.

To estimate the frequency of false positives from the technical background noise, we generated sequencing data for biological replicates (n = 21) using PBL DNA from six patients. One of the replicates from each patient was paired with the other as a mock-matched plasma sample and was processed for variant detection as described in Materials and Methods. No variants were detected out of a total of 21 replicates, indicating a minimal false discovery rate due to technical background. Collectively, our results show that targeted deep sequencing of plasma DNA in biopsy-free situations is a feasible means of detecting tumor-specific somatic variants across target regions.

There are limitations to using FNA samples for genetic profiling as they do not sufficiently represent all regional subclonal events. Our data suggests that somatic profiling of mutations in plasma DNA in a biopsy-free manner compensates for the shortcomings of FNA samples, revealing intra-tumor heterogeneity. Based on mutations in plasma DNA in target regions (M_P/TR_) combined with M_P/FNA_ and M_P/TR-BF_, we were able to detect ctDNAs in 15 out of 17 pretreatment samples, suggesting there is a significant advantage to profiling broader genomic regions instead of KRAS hotspots.

### Monitoring tumor burden by measuring ctDNA

**Figure 2:**
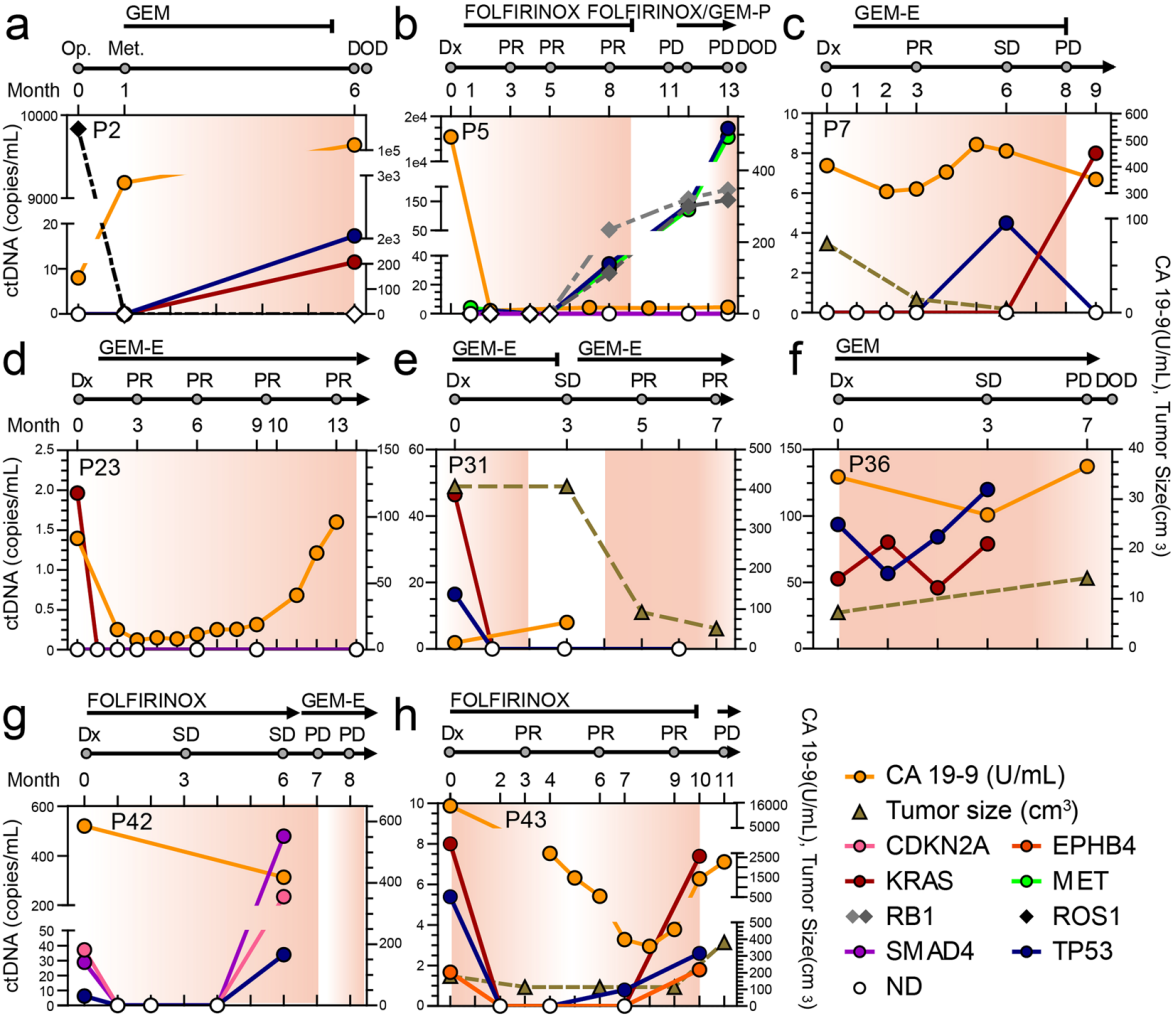
Monitoring of ctDNA in pancreatic cancer patients undergoing therapeutic interventions. The ctDNA levels estimated by M_P/TR_ were plotted on the left y-axis for eight patients (**a**–**h**). The entire list of M_P/TR_ is summarized in Supplementary Table 6. Chemotherapeutic agents administered to each patient and therapeutic response was evaluated based on the Response Evaluation Criteria In Solid Tumors are displayed at the top of the graph. CA 19-9 level (yellow solid line) and tumor size (dark-khaki dotted line) based on CT images were plotted against the right y-axis. CCRT, concurrent chemoradiation therapy; CR, complete response; DOD, dead of disease; Dx, diagnosis; E, erlotinib; GEM, gemcitabine; FU, fluorouracil; P, cisplatin; Met, metastasis; ND, not detected; Op, operation; PD, progressive disease; PR, partial response; SD, stable disease.

We next examined whether ctDNA levels correlated with patient clinical response to therapy and disease progression. First, we measured cancer antigen 19-9 (CA 19-9) and M_P/TR_ in consecutive blood draws using separate draws for each type of test from ten PDAC patients undergoing distinct treatment protocols. Except for two patients (P11 and P27), where variants were not detected in either primary or plasma samples, the data from the remaining eight patients is displayed in Fig. 2. Next, we examined if the M_P/TR_ frequencies and CA19-9 levels were indicative of changes in disease burden in these eight patients, in particular in terms of evaluation of complete response/partial response (CR/PR) and progressive disease (PD). M_P/TR_ were not detected in all five cases (P5, P7, P23, P31, and P43) when PR was determined and were elevated in all six cases (P2, P5, P7, P36, P42, and P43) when PD was observed. Therefore, these values positively correlated with disease burden in all 11 patients (Supplementary Table S7). Meanwhile, CA19-9 levels properly signaled a positive response in two out of four cases of PR and disease progression in four out of six cases of PD (Supplementary Table S7). These data suggest that ctDNA detected by M_P/TR_ is a better surrogate marker of patient response to chemotherapy and/or disease progression than CA19-9 in PDAC patients.

Among the eight patients, five patients (P5, P7, P23, P31, and P43) had computed tomography (CT) imaging data available more than three times during the tracing of the M_P/TR_ level. For three of these patients (P23, P31, and P43), significant decreases in the M_P/TR_ levels were followed by a PR evaluation (Fig. 2d,e,h). On the other hand, significantly detectable ctDNA levels and/or dramatic increases in the M_P/TR_ level were followed by disease progression in P5 and P43 patients (Fig. 2b,h). On average, alterations in M_P/TR_ frequencies were detected 2 months ahead of the CT imaging changes observed in these patients, suggesting that ctDNA measured by targeted deep sequencing is the earliest indicator of disease status. Taken together, these results suggest there is clinical utility for cfDNA sequencing in monitoring of PDAC patient clinical responses to therapy and disease progression.

**Figure 3:**
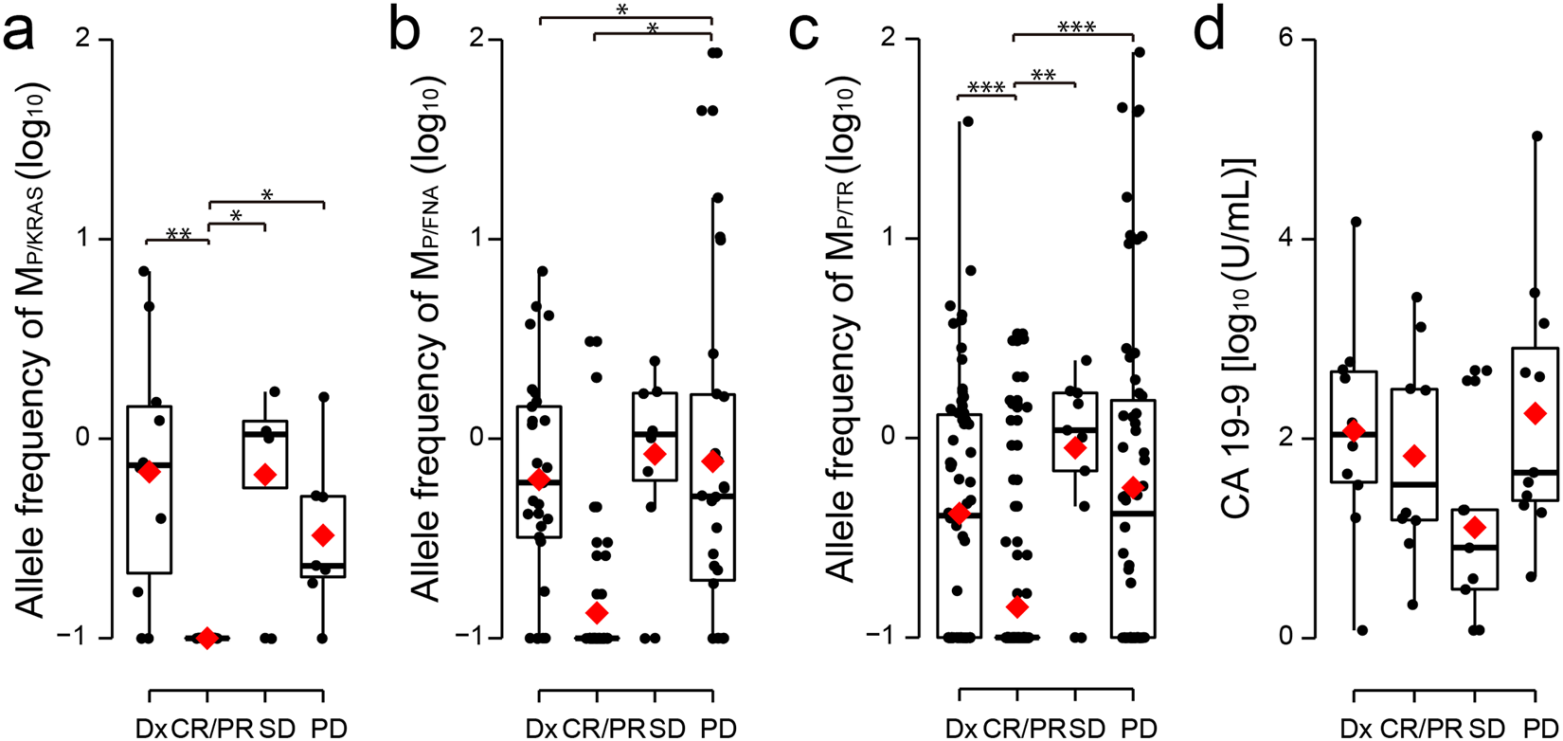
Correlation of allelic frequency of ctDNA and CA19-9 levels with patient response to therapy. The allelic frequencies of (**a**) M_P/KRAS_, (**b**) M_P/FNA_, and (**c**) M_P/TR_ were box-plotted based on evaluations of their near-time response to therapy and diagnosis. (**d**) CA 19-9 levels were box-plotted. All of the determined levels are presented on a logarithmic scale. In each box-plot, median and mean are indicated by a horizontal bar and red diamond, respectively. The level of statistical significance (ANOVA, LSD) is indicated as *P ≤ 0.05, **P ≤ 0.001, and ***P ≤ 0.00001. CR, complete response; Dx, diagnosis; PD, progressive disease; PR, partial response; SD, stable disease.

Next, to compare the allelic frequency of ctDNA with tumor burden, we divided our ctDNA data into four groups based on ctDNA at the time of 1) diagnosis (Dx) and when 2) CR/PR, 3) stable disease (SD), and 4) PD were noted (Fig. 3). The average allelic frequencies of ctDNA measured by all three different approaches varied among the groups, where the most significant was for M_P/TR_ (analysis of variance (ANOVA), least significant difference (LSD), p-value = 4.5 × 10^−9^, Fig. 3c) followed by M_P/KRAS_ (ANOVA, LSD, p-value = 0.0018, Fig. 3a). We noticed the allelic fractions of M_P/TR_ near the time of CR/PR were significantly lower than those at the time of Dx, SD, and PD (Fig. 3c). Because an increase in M_P/TR_ frequency was likely to precede determination of PD, we moved data points obtained less than 3 months before PD, regardless of what group they belonged to, into the PD group in order to take the time lag into account. When we made this adjustment, the statistical significance increased further, supporting this finding (ANOVA, LSD, p-value = 1.2 × 10^−12^, Supplementary Fig. S2). Meanwhile, CA 19-9 levels were not significantly different among patients with different disease statuses (ANOVA, LSD, p-value = 0.13, Fig. 3d).

**Figure 4:**
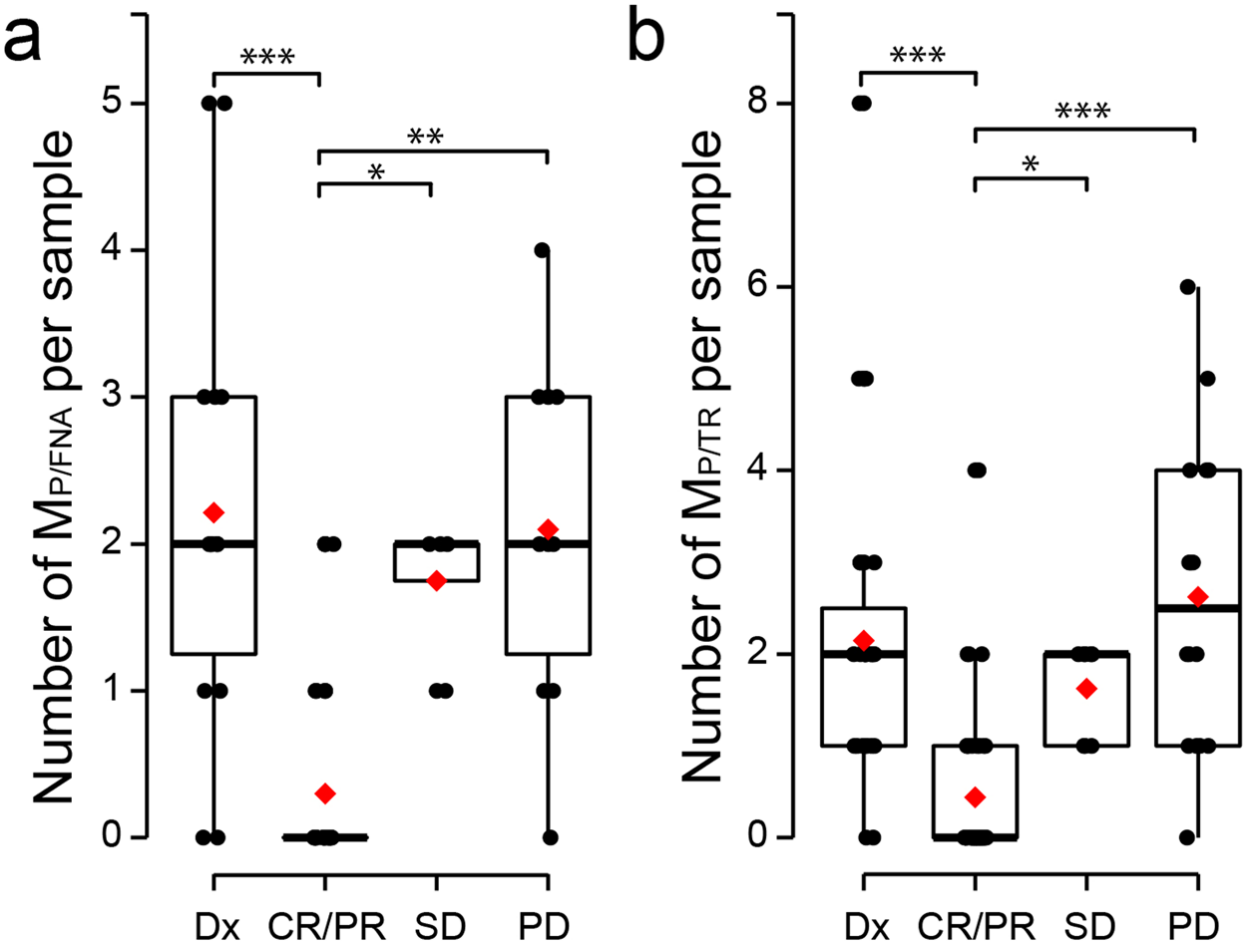
Number of mutations in plasma DNA. The (**a**) M_P/FNA_ and (**b**) M_P/TR_ in each sample were box-plotted according to evaluations of near-time therapy response and diagnosis. The level of statistical significance (ANOVA, LSD) is indicated as *P ≤ 0.05, **P ≤ 0.001, and ***P ≤ 0.00001. CR, complete response; Dx, diagnosis; PD, progressive disease; PR, partial response; SD, stable disease.

Because the allelic frequencies in ctDNA varied according to disease status, we also quantified the average number of variants detected per sample (Fig. 4). We noticed the number of variants detected differed significantly among patients with different disease statuses when analyzing M_P/FNA_ (Fig. 4a, ANOVA, LSD, p-value = 1.8 × 10^−5^) and M_P/TR_ (Fig. 4b, ANOVA, LSD, p-value = 5.7 × 10^−8^). Similar to the diminished allelic frequencies of ctDNA in the CR/PR group, the number of M_P/FNA_ and M_P/TR_ had significantly decreased at the time of CR/PR compared to the other groups. To exclude the possibility that the low allelic frequencies and numbers of M_P/TR_ in the CR/PR group were due to technical artifacts, we compared sequencing metrics among the groups. We found no differences in these parameters, thus ruling out the aforementioned possibility (Supplementary Figs S3 and S4).

**Figure 5:**
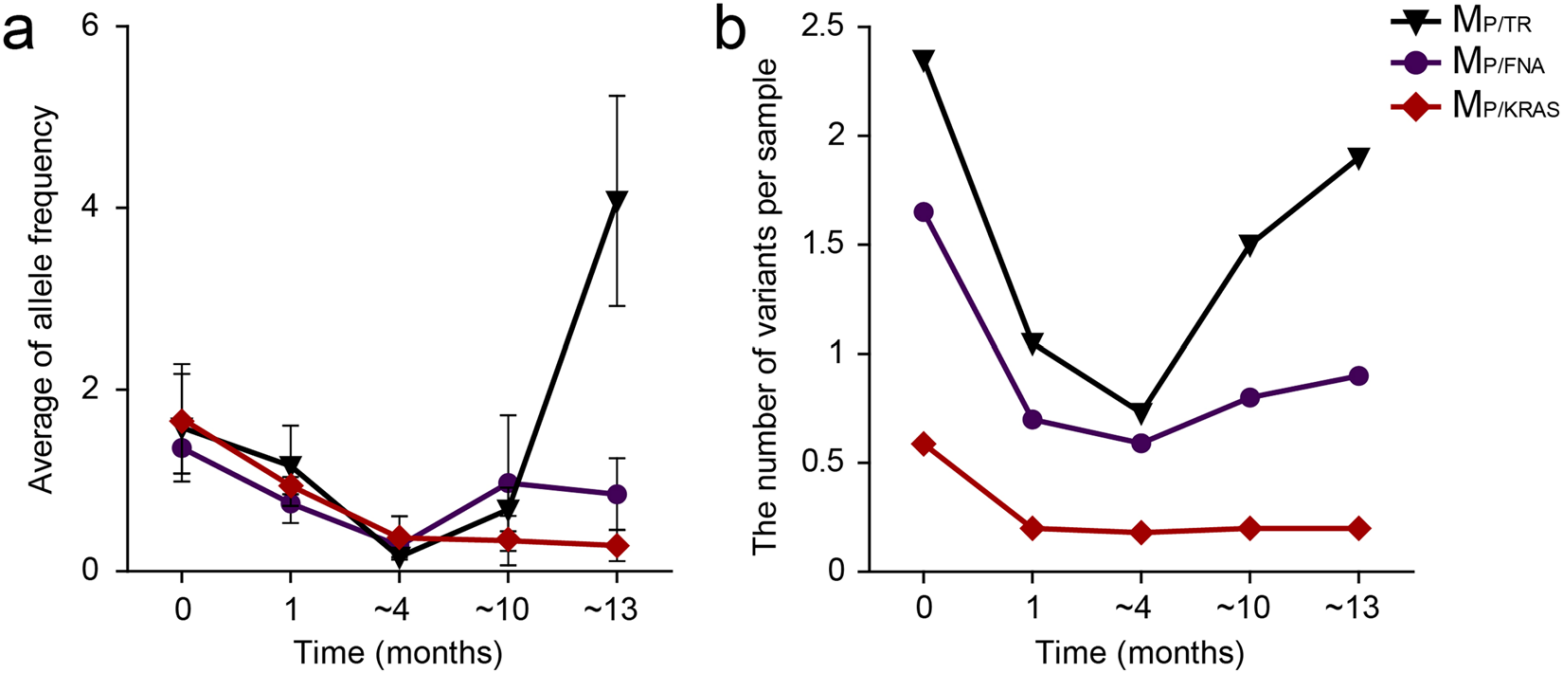
Levels of ctDNA based on treatment duration. (**a**) Allelic frequencies and (**b**) numbers of mutations were averaged and plotted based on duration of chemotherapy.

Noticeably, the allelic frequency and number of M_P/TR_ started to decrease immediately after treatment was initiated and were lowest around 4 months of post-treatment (Fig. 5). Thereafter, both the allelic frequency and number of M_P/TR_ significantly increased as treatment duration increased (ANOVA, LSD, p-value = 0.008 for Fig. 5a; ANOVA, LSD, p-value = 0.004 for Fig. 5b). These data are also consistent with our results showing that M_P/TR_ correlates with evaluation of response to therapy, as PR was most frequently observed around up to 4 months following treatment with chemotherapeutics.

Overall, our results indicate that M_P/TR_ is a better representation of real-time disease status based on either allele frequency and/or number of mutations than M_P/KRAS_ and M_P/FNA_. Taken together, our results strongly suggest that the allelic and/or M_P/TR_ frequencies are good indicators of real-time disease status in PDAC patients, providing information not only concerning the presence of disease, but also whether there is a positive response to therapy, even when it is a partial response.

## Discussion

In this study, we evaluated ctDNA-based approaches for detecting PDAC. Specifically, we profiled (1) somatic variants in KRAS hotspots (M_P/KRAS_), (2) patient-specific mutations in tissue samples (M_P/FNA_), and (3) broad genomic target regions (M_P/TR_). Of these approaches, we found comprehensive analysis of ctDNA across broad genomic target regions was the most sensitive at detecting PDACs and accurately monitoring tumor burden. Furthermore, analysis of ctDNA might overcome the limitation in variant detection in FNA samples due to tumor purity and/or intra-tumor heterogeneity.

Among the 17 pretreatment plasma samples, we detected ctDNA in ten, twelve, and fifteen samples when profiling M_P/KRAS_, M_P/FNA_, and M_P/TR_, respectively, indicating an increased sensitivity of cancer detection by analyzing cfDNA across broad genomic target regions. Moreover, this improved sensitivity in ctDNA detection enhanced tumor monitoring by longitudinal cfDNA analysis. For instance, in patients P5 and P42, although a KRAS mutation was not detected in pretreatment samples or any subsequent peri/post-treatment samples, multiple independent variants in other genes were detected whose levels consistently correlated with tumor burden (Fig. 2b,g). In patient P2, ROS1 p.I1967V levels in plasma DNA dramatically decreased after a surgical operation, which is indicative of tumor removal; however, this was not reflected in the M_P/KRAS_ and M_P/FNA_ (Fig. 2a).

Despite its advantages, interrogation of broader genomic regions may result in a higher frequency of false positives, especially when performed in a biopsy-free manner. In fact, several studies have utilized digital PCR in parallel with targeted deep sequencing, which can complement or cross-validate each other for detecting ctDNA in various types of cancers^8,35,36^. Furthermore, the size of the target regions queried has been taken into account for optimally balancing the sensitivity and specificity of detecting ctDNA in previous studies^9,32^. To minimize these false positives, we used stringent filtering steps when calling M_P/TR-BF_. Then, we evaluated if the filtering steps during variant calling were adequate for reducing the rate of false discoveries. By analyzing duplicated PBL genomic DNA sequencing data, we found that false positives due to technical background were minimal, as described in the Results section. In addition, we validated some of the M_P/TR-BF_ not detected in FNA specimens by ddPCR, which indicated using the variant detection method in a biopsy-free manner was stringent enough to remove most false positives.

The sensitivity of variant detection is significantly dependent on the depth of unique coverage, although this is not the sole determinant. Since the method to call M_P/TR-BF_ did not use a threshold of the MAF, we calculated the lowest MAF of detectable M_P/TR-BF_, which varied depending on the depth of unique coverage (Supplementary Fig. S5a). In addition, we examined the allele-specific background error rates across the target regions (Supplementary Fig. S5b) because a practical limit of detection is also imposed by errors introduced during sample preparation and sequencing. However, due to elevated background error rates, only 1.58% of alternative alleles required more supporting reads than the threshold (n = 8) in the calling algorithm for M_P/TR-BF_ (Supplementary Fig. S5c). These data indicated that the lowest theoretical MAF was applicable to >98% of the alternative alleles. We next calculated the lowest theoretical MAF of M_P/TR-BF_ depending on the amount of input DNA, which was correlated with the depth of unique coverage as shown in Supplementary Fig. S6a. The average threshold for calling M_P/TR-BF_ varied in the range of 0.24–0.70% depending on the input DNA amount (7.8–102 ng, except for one outlier of 212 ng) used for library construction in this study.

When we sequenced cfDNA reference DNA (50 ng) from Horizon, which contains hotspot mutations at a frequency of 0.1%, 1%, and 5%, we were able to detect all six SNVs at the 1% frequency but none at the 0.1% frequency using the M_P/TR-BF_ calling method. The data were consistent with the estimated lowest theoretical MAF of M_P/TR-BR_. Although the number of variants tested was small and the interval of tested frequency was wide, the data showed that the MAF detection limit of the method was around 1% or less.

Because we applied stringent filtering steps to minimize the false discovery rate when detecting M_P/TR-BF_, the variant calling method might compromise the analytic detection sensitivity. Combining this method with M_P/FNA_ allowed detection at a high sensitivity by taking advantage of patient-specific mutations in tissue samples. Therefore, we could compensate for the compromised detection sensitivity while interrogating somatic tumor variants across broad genomic regions.

Molecular barcoding has recently been shown to minimize technical background noise and overcome the loss of unique reads due to fragment identification collision during deduplication^26^. However, this method was not utilized in this study. Therefore, modification to include molecular barcoding is anticipated to further improve the specificity and sensitivity of targeted deep sequencing of broad genomic regions to detect ctDNA.

As the profiles of genetic alterations in PDAC may allow physicians to determine tailored therapeutics by offering useful information on probable clinical outcomes, genetic testing using tumor samples is becoming increasingly important^37^. Although genetic tests for PDAC are often conducted using endoscopic ultrasound (EUS)-guided FNA samples^38,39^, FNA specimens often have low cellularity and contain a significant fraction of normal stromal cells^24^. Consequently, genetic tests using FNA samples frequently suffer from small input amounts of DNA that contain only a small fraction of tumor DNA, which affects the analytic detection sensitivity of these tests^40^. We also detected KRAS mutations (allele frequency ≥4%) in 76.5% of FNA specimens, which is somewhat lower than KRAS mutation-positive rates observed in surgical specimens. Additionally, FNA samples might not thoroughly represent various subclonal populations in heterogeneous tumor masses. As our results included tumor variants that were not detected in FNA samples, but were detected in the corresponding plasma samples, the limitations of using FNA samples for genetic testing may be at least partly overcome by detecting ctDNA by analyzing broad genomic regions.

There are obvious hurdles for translating targeted sequencing of cfDNA into clinical tests for PDAC. First, the higher cost of targeted deep sequencing might outbalance the benefit of profiling genetic alterations across broad genomic regions at this time. However, the recent dramatic reduction in sequencing costs implies that the cost-effectiveness of this method is likely to increase in the near future. Second, this study is a pilot test that analyzed only a small number of cases with the heterogeneous treatments, although we revealed the advantages of analyzing broad genomic regions to detect ctDNA in PDAC patients. A large cohort study is required to evaluate the clinical utility of this method for PDACs.

In summary, our results suggest that using targeted deep sequencing of broad genomic regions to detect ctDNA has diagnostic advantages, even for PDACs that typically harbor a KRAS hotspot mutation.

## Materials and Methods

### Patient samples

The institutional review board at the Samsung Medical Center approved this present study (IRB number 2014-04-048-009) and all the methods were carried out in accordance with the approved guidelines. Written informed consent was obtained from all subjects. Newly diagnosed PDAC patients who had undergone EUS-guided FNA were enrolled in and underwent blood draws for cfDNA testing. The pretreatment (i.e., before treatment) blood draw of participants was collected at the time of diagnosis.

### Plasma and PBL sample preparation

Whole blood samples were collected in Cell-Free DNA™ BCT tubes (Streck Inc., Omaha, NE, USA). Plasma was prepared using 3 centrifugation steps with increasing centrifugal force: 840 × *g* for 10 min, 1040 × *g* for 10 min, and then 5000 × *g* for 10 min at room temperature. PBLs were collected from the initial centrifugation step. Collected plasma and PBL samples were stored at −80 °C until DNA extraction.

### DNA sample preparation

PBL genomic DNA was isolated using a QIAamp DNA mini kit (Qiagen, Santa Clarita, CA, USA). Plasma DNA was obtained from 2 to 5 mL of plasma using a QIAamp Circulating Nucleic Acid Kit (Qiagen). An AllPrep DNA/RNA Mini Kit (Qiagen) was used to purify genomic DNA from FNA tissues. The concentration, purity, and fragment size of DNA were assessed as previously described^33,41^. In addition, the Horizon cfDNA reference standard set (HD780, Horizon Discovery Group plc, Cambridge, UK) was obtained to evaluate the performance of variant detection. SNVs in the reference materials included EGFR L858R, EGFR T790M, KRASG12D, NRAS Q61K, NRAS A59T, and PIK3CA E545K.

### Library preparation

Purified genomic DNA was sonicated (7 min, 0.5% duty, intensity of 0.1, and 50 cycles/burst) into 150–200 bp fragments using a Covaris S2 (Covaris Inc. Woburn, MA, USA). The FNA sample libraries were constructed using the SureSelect XT reagent kit, HSQ (Agilent Technologies) according to the manufacturer’s instructions. The PBL and plasma DNA libraries were created using a KAPA Hyper Prep Kit (Kapa Biosystems, Woburn, MA, USA) as described previously^33,41^. When constructing the sequencing libraries, 200 ng of PBL DNA and 37.12 ng of plasma DNA were used on average. Briefly, after end repair and A-tailing according to the manufacturer’s protocol, we performed adaptor ligation at 4 °C overnight using a pre-indexed PentAdapter™ (PentaBase ApS, Denmark). After amplification using 9 PCR cycles, the library was analyzed for quantity and fragment size distribution and then underwent multiplexing hybrid selection to enrich for targets. Hybrid selection was performed using customized RNA baits that targeted ~499 kb of the human genome, including exons from 83 cancer-related genes (Supplementary Table 1). Up to 8 purified libraries were pooled and a total of 750 ng of each pooled library was used for hybrid selection reactions. Target enrichment was performed according to the SureSelect (Agilent Technologies) bait hybridization protocol except the blocking oligonucleotide was replaced with the IDT xGen blocking oligonucleotide (IDT, Santa Clara, CA, USA) as the pre-indexed adapter. After target enrichment, the captured DNA fragments were amplified using 13 cycles of PCR with oligonucleotides P5 and P7.

### Sequencing and data processing

Based on DNA concentration and average fragment size, each library was diluted to a concentration of 2 nM and pooled in equal volumes. The libraries were denatured using 0.2 N NaOH, diluted to 20 pM in hybridization buffer (Illumina, San Diego, CA, USA), and then subjected to cluster amplification according to the manufacturer’s protocol (Illumina). Flow cells were sequenced in the 100 bp paired-end mode using HiSeq. 2500 v3 Sequencing-by-Synthesis Kits (Illumina) and then analyzed using RTA v.1.12.4.2 or later. All of the raw data were aligned to the hg19 human reference and BAM files were created using BWA-mem (v0.7.5)^42^. SAMTOOLS (v0.1.18)^43^, Picard (v1.93), and GATK (v3.1.1)^44^ were used for sorting SAM/BAM files, local realignment, and duplicate markings, respectively. We filtered reads to remove duplicates, discordant pairs, and off-target reads as previously described^33^.

### SNV detection in FNA samples and statistical tests for the SNVs in the matched plasma

For FNA biopsy specimens, somatic SNVs were detected using MuTect 1.1.4^45^ and Varscan2^46^ with matched germline (i.e., PBL) samples. For Varscan2, the default parameter values were used with previously described modifications^9^. Somatic SNVs identified by at least one method were retained if they were present at a frequency lower than 0.5% in the matched PBL sample and higher than 4% in the tumor sample. Somatic SNVs found in the FNA samples (M_FNA_) were listed and tested for their presence in the paired plasma samples as described previously^9^ in our recent study^34^. After background alleles in each sample were adjusted based on position-specific error rates, it was determined if the allelic frequency of a given SNV fell within the 95th percentile of the adjusted background alleles. The depth of unique coverage, strand bias, and supporting read count were also considered for SNV detection.

### Biopsy-free SNV (M_P/TR-BF_) identification in plasma DNA

A method from previous studies^8,9^ was modified slightly to identify somatic SNVs in plasma samples as described in our recent study^34^. First, positions with a strand bias under 0.9 and total read depth over 500 were considered for analysis. All non-reference alleles were subjected to Phred quality filtering using a threshold Q of 30. Non-reference alleles present at a frequency lower than 0.5% in the matched germline DNA were subjected to the binomial test to determine if a non-reference allele was significantly more abundant in plasma DNA than the matched germline DNA. Multiple testing adjustments were made using the Bonferroni correction. Next, z-tests were performed to compare the frequencies of nonreference alleles to their background allele frequency distribution in other plasma DNA samples. For comparison, a background allele frequency distribution was generated by selecting non-reference alleles in plasma DNA present at a frequency <2.5% in the paired tumor and <0.5% in the paired germline DNA with a sufficient total depth (≥250× in tumor tissue, ≥500× in PBL, and ≥500× in plasma). In addition, the following filters were applied: (1) candidate alleles with less than eight supporting reads, (2) all candidates with an allele frequency <20% when there were two or more candidates within any 10 bp window, and (3) candidates with a Bonferroni adjusted p-value higher than 10^−18^ from the z-test were discarded. To remove potential false positives due to cross-contamination among multiplexed samples, we excluded SNV candidates if they were found as germline single nucleotide polymorphisms (SNPs) in other samples processed in the same lane of a sequencing flow cell. Nonsynonymous, stop-gain, stop-loss, and splicing-disrupting variants were listed as the final positive calls.

### Droplet digital PCR validation

Mutant and wild-type alleles in plasma samples were quantified using the QX200 Droplet Digital PCR System (Bio-Rad, Hercules, CA, USA). TaqMan assays for KRAS p.G12D/G12V were obtained from Bio-Rad (PrimePCR ddPCR Mutation Assay) and RB1 p.R251* and ROS1 p.I1967V assays were custom generated by TaqMan SNP Genotyping Assays (Thermo Fisher Scientific, Waltham, MA, USA). The concentrations of wild-type and mutant DNA (copies per µl) in each sample were calculated using the manufacturer’s software and the concentrations in plasma (copies per mL) were derived as described in van Ginkel, J.H *et al*.^47^.

### Statistical analysis

To evaluate whether the means between multiple groups were significantly different, we used one-way ANOVA with LSD post-hoc analysis. For all of the tests, statistical significance was set at 5% and reported as two-tailed p-values. Statistical analyses were carried out using PASW Statistics for Windows version Release 18.0.0 (formerly SPSS, IBM Corporation, Armonk, New York).

### Accession codes

Raw sequencing data were deposited in the Sequence Read Archive with accession number SRP097813.

## Electronic supplementary material

Supplementary Information

## References

[1] Makohon-Moore A, Iacobuzio-Donahue CA (2016). Pancreatic cancer biology and genetics from an evolutionary perspective. Nat Rev Cancer.

[2] Kubota M (2000). Cancer chemotherapy and somatic cell mutation. Mutat Res.

[3] Ryan DP, Hong TS, Bardeesy N (2014). Pancreatic adenocarcinoma. N Engl J Med.

[4] Lennon, A. M. & Goggins, M. Diagnostic and Therapeutic Response Markers. In Pancreatic Cancer 675-701 (Springer New York, New York, NY, 2010).

[5] Takai E, Yachida S (2016). Circulating tumor DNA as a liquid biopsy target for detection of pancreatic cancer. World J Gastroenterol.

[6] Pietrasz D (2017). Plasma Circulating Tumor DNA in Pancreatic Cancer Patients Is a Prognostic Marker. Clin Cancer Res.

[7] Bettegowda C (2014). Detection of circulating tumor DNA in early- and late-stage human malignancies. Sci Transl Med.

[8] Takai E (2015). Clinical utility of circulating tumor DNA for molecular assessment in pancreatic cancer. Sci Rep.

[9] Newman AM (2014). An ultrasensitive method for quantitating circulating tumor DNA with broad patient coverage. Nat Med.

[10] Murtaza M (2015). Multifocal clonal evolution characterized using circulating tumour DNA in a case of metastatic breast cancer. Nat Commun.

[11] Remon J (2017). Osimertinib benefit in EGFR-mutant NSCLC patients with T790M-mutation detected by circulating tumour DNA. Ann Oncol.

[12] Phallen, J. *et al*. Direct detection of early-stage cancers using circulating tumor DNA. *Sci Transl Med* **9** (2017). 10.1126/scitranslmed.aan2415 PMC6714979 28814544

[13] Wan, J. C. *et al*. Liquid biopsies come of age: towards implementation of circulating tumour DNA. *Nat Rev Cancer* (2017). 10.1038/nrc.2017.7 28233803

[14] Diehl F (2008). Circulating mutant DNA to assess tumor dynamics. Nat Med.

[15] Oxnard GR (2016). Association Between Plasma Genotyping and Outcomes of Treatment With Osimertinib (AZD9291) in Advanced Non-Small-Cell Lung Cancer. J Clin Oncol.

[16] Sacher AG (2016). Prospective Validation of Rapid Plasma Genotyping for the Detection of EGFR and KRAS Mutations in Advanced Lung Cancer. JAMA Oncol.

[17] Li M, Diehl F, Dressman D, Vogelstein B, Kinzler KW (2006). BEAMing up for detection and quantification of rare sequence variants. Nat Methods.

[18] Tjensvoll K (2016). Clinical relevance of circulating KRAS mutated DNA in plasma from patients with advanced pancreatic cancer. Mol Oncol.

[19] Dabritz J, Preston R, Hanfler J, Oettle H (2009). Follow-up study of K-ras mutations in the plasma of patients with pancreatic cancer: correlation with clinical features and carbohydrate antigen 19-9. Pancreas.

[20] Brychta N, Krahn T, von Ahsen O (2016). Detection of KRAS Mutations in Circulating Tumor DNA by Digital PCR in Early Stages of Pancreatic Cancer. Clin Chem.

[21] Ako S (2017). Utility of serum DNA as a marker for KRAS mutations in pancreatic cancer tissue. Pancreatology.

[22] Cohen, J. D. *et al*. Detection and localization of surgically resectable cancers with a multi-analyte blood test. *Science* (2018). 10.1126/science.aar3247 PMC6080308 29348365

[23] Earl J (2015). Circulating tumor cells (Ctc) and kras mutant circulating free Dna (cfdna) detection in peripheral blood as biomarkers in patients diagnosed with exocrine pancreatic cancer. BMC Cancer.

[24] Cancer Genome Atlas Research Network. Electronic address, a.a.d.h.e. & Cancer Genome Atlas Research, N. Integrated Genomic Characterization of Pancreatic Ductal Adenocarcinoma. Cancer Cell 32, 185–203 e13 (2017). 10.1016/j.ccell.2017.07.007 PMC5964983 28810144

[25] Forshew T (2012). Noninvasive identification and monitoring of cancer mutations by targeted deep sequencing of plasma DNA. Sci Transl Med.

[26] Newman AM (2016). Integrated digital error suppression for improved detection of circulating tumor DNA. Nat Biotechnol.

[27] Dawson SJ (2013). Analysis of circulating tumor DNA to monitor metastatic breast cancer. N Engl J Med.

[28] De Mattos-Arruda L (2014). Capturing intra-tumor genetic heterogeneity by de novo mutation profiling of circulating cell-free tumor DNA: a proof-of-principle. Ann Oncol.

[29] Murtaza M (2013). Non-invasive analysis of acquired resistance to cancer therapy by sequencing of plasma DNA. Nature.

[30] Vandekerkhove, G. R. *et al*. Circulating tumor DNA reveals clinically-actionable somatic genome of metastatic bladder cancer. *Clin Cancer Res* (2017). 10.1158/1078-0432.CCR-17-1140 28760909

[31] Abbosh C (2017). Phylogenetic ctDNA analysis depicts early-stage lung cancer evolution. Nature.

[32] Cohen JD (2018). Detection and localization of surgically resectable cancers with a multi-analyte blood test. Science.

[33] Park G (2017). Characterization of background noise in capture-based targeted sequencing data. Genome Biol.

[34] Kim JY (2017). Circulating tumor DNA shows variable clonal response of breast cancer during neoadjuvant chemotherapy. Oncotarget.

[35] Siravegna G (2015). Clonal evolution and resistance to EGFR blockade in the blood of colorectal cancer patients. Nat Med.

[36] Leary RJ (2010). Development of personalized tumor biomarkers using massively parallel sequencing. Sci Transl Med.

[37] Schneider G, Schmidt-Supprian M, Rad R, Saur D (2017). Tissue-specific tumorigenesis: context matters. Nat Rev Cancer.

[38] Eloubeidi MA (2003). Endoscopic ultrasound-guided fine needle aspiration biopsy of patients with suspected pancreatic cancer: diagnostic accuracy and acute and 30-day complications. Am J Gastroenterol.

[39] Eloubeidi MA (2007). A prospective evaluation of an algorithm incorporating routine preoperative endoscopic ultrasound-guided fine needle aspiration in suspected pancreatic cancer. J Gastrointest Surg.

[40] Giovannini M (2006). Endoscopic ultrasound elastography: the first step towards virtual biopsy? Preliminary results in 49 patients. Endoscopy.

[41] Chung J (2016). The minimal amount of starting DNA for Agilent’s hybrid capture-based targeted massively parallel sequencing. Sci Rep.

[42] Li H, Durbin R (2010). Fast and accurate long-read alignment with Burrows-Wheeler transform. Bioinformatics.

[43] Li H (2009). The Sequence Alignment/Map format and SAMtools. Bioinformatics.

[44] McKenna A (2010). The Genome Analysis Toolkit: a MapReduce framework for analyzing next-generation DNA sequencing data. Genome Res.

[45] Cibulskis K (2013). Sensitive detection of somatic point mutations in impure and heterogeneous cancer samples. Nat Biotechnol.

[46] Koboldt DC (2012). VarScan 2: somatic mutation and copy number alteration discovery in cancer by exome sequencing. Genome Res.

[47] van Ginkel JH, Huibers MMH, van Es RJJ, de Bree R, Willems SM (2017). Droplet digital PCR for detection and quantification of circulating tumor DNA in plasma of head and neck cancer patients. BMC Cancer.

