# Supplementary material for: Utility of targeted deep sequencing for detecting circulating tumor DNA in pancreatic cancer patients
Source: Sci Rep. 2018 Aug 2;8:11631. doi: 10.1038/s41598-018-30100-w (PMC6072791; doi:10.1038/s41598-018-30100-w)
Supplement: Supplementary file 1 — Supplementary Information [file 41598_2018_30100_MOESM1_ESM.docx]

**Supplementary Information**

**Utility of targeted deep sequencing for detecting circulating tumor DNA in pancreatic cancer patients**

Gahee Park^1,2#^, Joo Kyung Park^3#^, Dae-Soon Son^1^, Seung-Ho Shin^1,4^, Yeon Jeong Kim^1^, Hyo-Jeong Jeon^1^, Jae Lee^1^, Woong-Yang Park^1,4,5^*, Kwang Hyuck Lee^3,4^*, and Donghyun Park^1^*

^1^ Samsung Genome Institute, Samsung Medical Center, Seoul 06351, Korea

^2^ Department of Biomedical Sciences, Seoul National University College of Medicine, Seoul 03080, Korea

^3^ Division of Gastroenterology, Department of Medicine, Samsung Medical Center, Sungkyunkwan University School of Medicine, Seoul 06351, Korea

^4^ Department of Health Sciences and Technology, Samsung Advanced Institute for Health Sciences & Technology, Sungkyunkwan University, Seoul 06351, Korea

^5^ Department of Molecular Cell Biology, Sungkyunkwan University School of Medicine, Suwon 16419, Korea

^#^ These authors contributed equally to this work.

* Corresponding author.

**CONTENTS**

- Supplementary Figure S1 3
- Supplementary Figure S2 4
- Supplementary Figure S3 5
- Supplementary Figure S4 6
- Supplementary Figure S5 7
- Supplementary Figure S6 8
- Supplementary Table S1 9
- Supplementary Table S2 15
- Supplementary Table S3 16
- Supplementary Table S4 20
- Supplementary Table S5 23
- Supplementary Table S6 25
- Supplementary Table S7 31


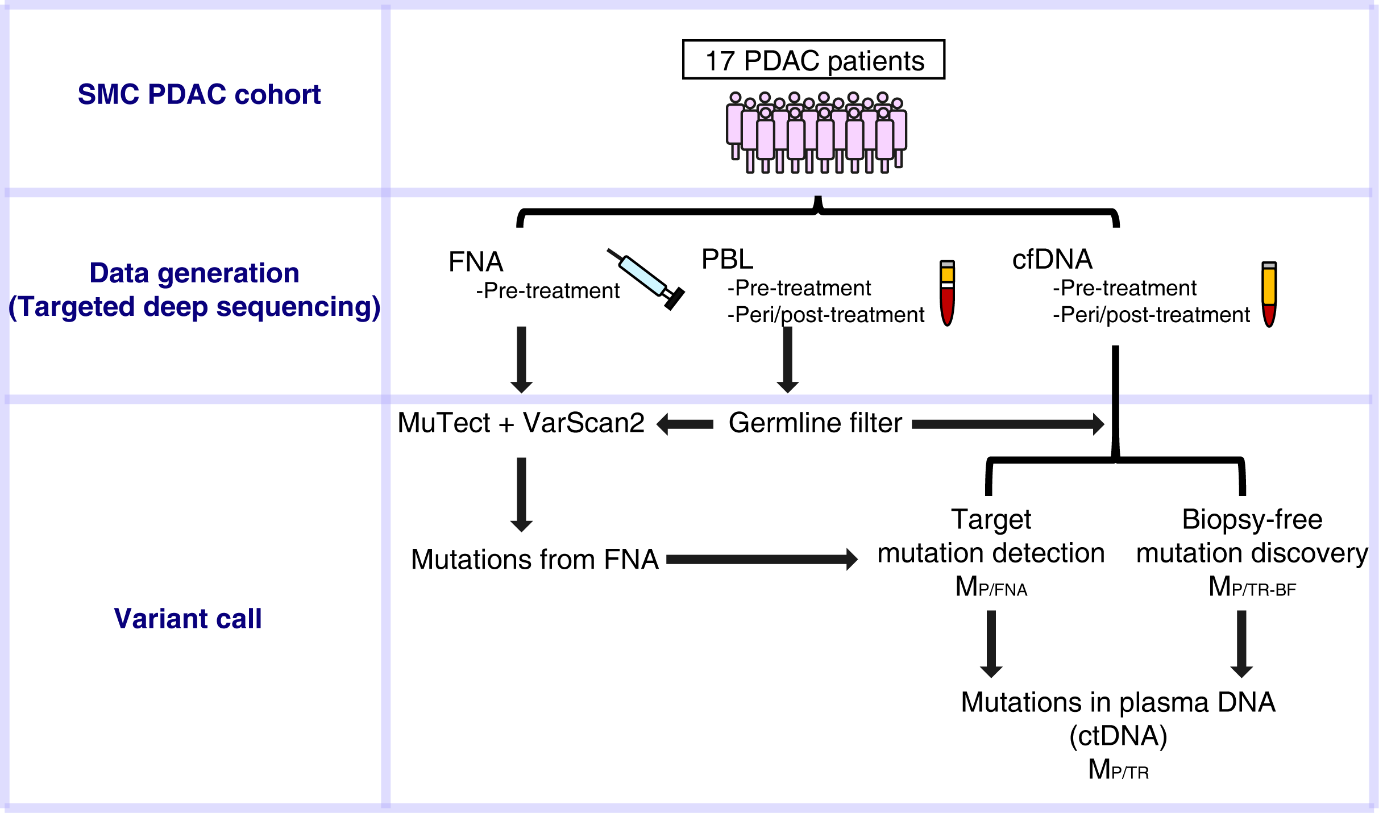


**Supplementary Figure S1.** Schematic analytical procedure in this study.


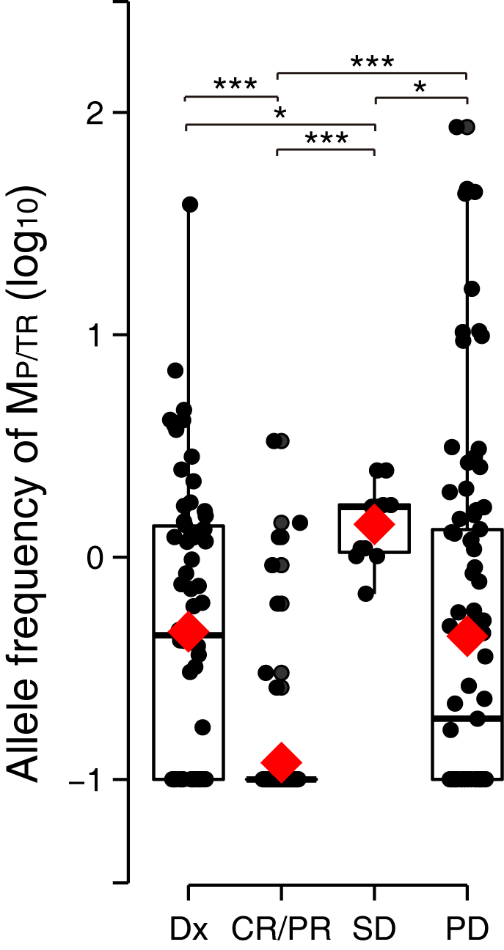


**Supplementary Figure S2.** The box-plot of the allele frequencies of M_P/TR_ in Figure 2 was modified by moving data points obtained 3 months before PD evaluation into the PD group. All of the determined levels were displayed on a logarithmic scale. In each box-plot, median and mean are indicated by horizontal bar and red diamond, respectively. The level of statistical significance (ANOVA, LSD) is indicated by the asterisks; * P ≤ 0.05, ** P ≤ 0.001, and *** P ≤ 0.00001. Dx, diagnosis; CR, complete response; PR, partial response; SD, stable disease; PD, progressive disease.

**
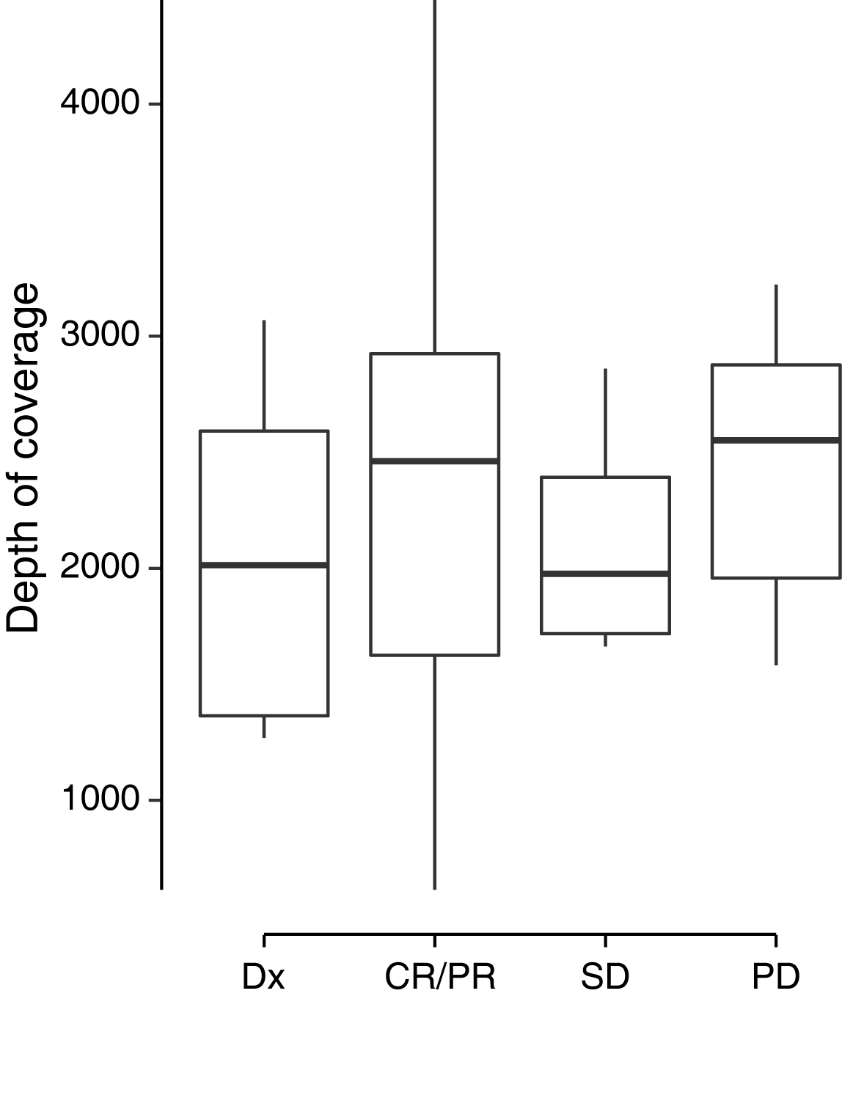
**

**Supplementary Figure S3.** Mean depth of unique coverage for cfDNA sequencing data was box-plotted depending on their near-time therapy response evaluation as well as diagnosis. Dx, diagnosis; CR, complete response; PR, partial response; SD, stable disease; PD, progressive disease.


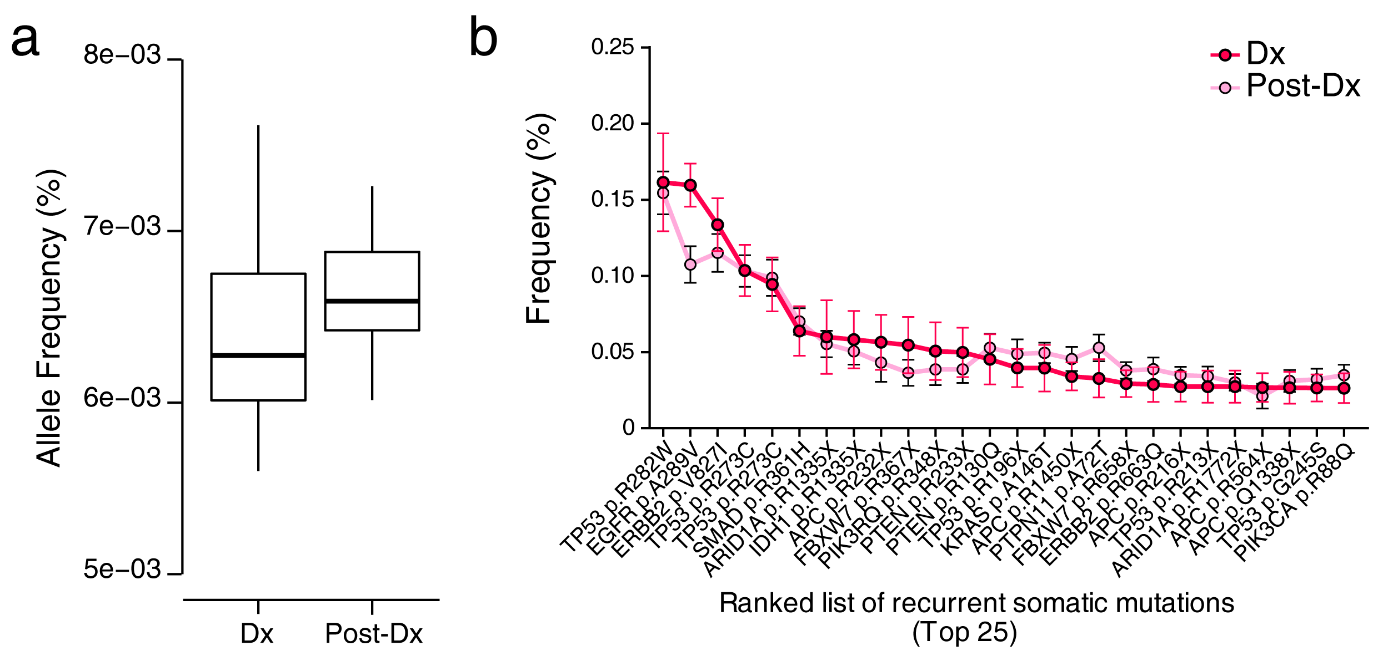


**Supplementary Figure S4.** (a) The frequency of background error across the entire target region was calculated for each sample and compared between diagnosis (Dx) and after diagnosis (post-Dx). There was no significant difference among the groups (ANOVA, LSD, p = 0.57). (b) The average background error rates in recurrent hotspots were compared between the groups. The error rates were sorted in decreasing order and top 25 are displayed.

**
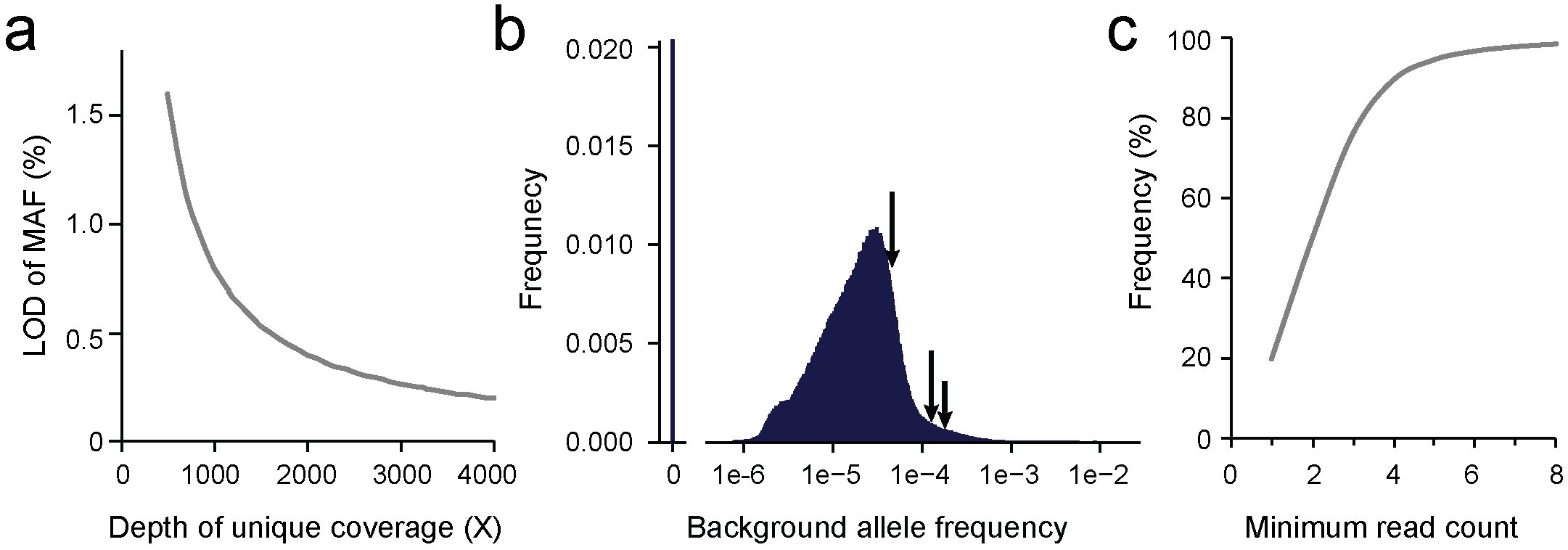
**

**Supplementary Figure S5.** (a) The lowest theoretical minor allele frequencies (MAFs) of M_P/TR-BR_ plotted according to the depth of unique coverage. (b) The average background error rates were calculated and their distribution was plotted for each non-reference allele across the entire target region. The arrows indicate the 50^th^, 90^th^, and 95^th^ percentile allele frequencies. (c) The minimal count of supporting reads to be discriminated from allele-specific background noise (p < 10^-18^ from the z-test) was calculated for each non-reference allele. With a given number of supporting reads, the cumulative fraction of non-reference alleles that could be discriminated from background noise and considered to be a variant candidate was plotted.

**
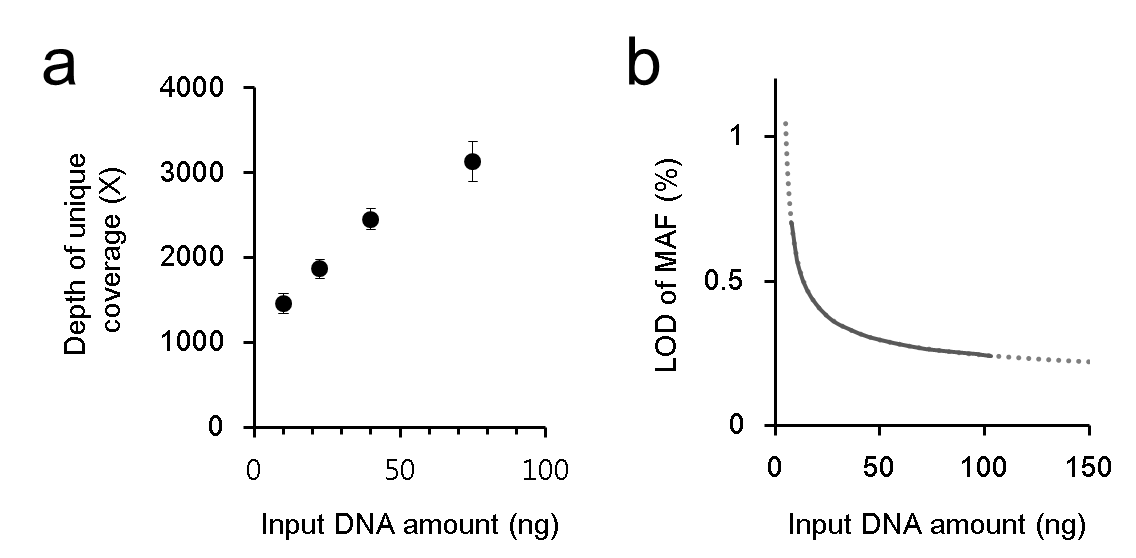
**

**Supplementary Figure S6.** Effects of the amount of input DNA used for library construction on (a) the depth of unique coverage and (b) the lowest minor allele frequencies (MAFs) of M_P/TR-BR_. (a) The mean depth of unique coverage was averaged from samples in each bin. The error bars indicate s.e.m. (b) The lowest MAFs depending on the input DNA amounts are plotted along the dotted line. The interval of input DNA amounts used in this study is marked as a solid line.

| **Supplementary Table S1. Summary of sequencing metrics** | | | | | | | | | |
| --- | --- | --- | --- | --- | --- | --- | --- | --- | --- |
| **Sample ID** | **DNA mass used for library (ng)** | **Volume of plasma used for library (mL)** | **Total**  **read** | **Mapped**  **read** | **Dedup**  **read** | **Duplication rate**  **(%)** | **On target bases** | **On target**  **(%)** | **Mean target coverage** |
| FNA_P2 | 200 |  | 14548348 | 13958921 | 11843833 | 15.2 | 508659621 | 43.0 | 1476.6 |
| FNA_P5 | 200 |  | 10617084 | 10177953 | 8117185 | 20.3 | 379944614 | 46.8 | 811.2 |
| FNA_P7 | 200 |  | 10076588 | 9518333 | 8022465 | 15.7 | 370154631 | 46.2 | 790.3 |
| FNA_P10 | 200 |  | 9751728 | 9319978 | 7973312 | 14.5 | 396407907 | 49.8 | 846.4 |
| FNA_P11 | 200 |  | 9889198 | 9374543 | 8127353 | 13.3 | 378571079 | 46.6 | 808.3 |
| FNA_P21 | 200 |  | 37150622 | 35457980 | 32268856 | 9.0 | 1184986811 | 36.7 | 949.4 |
| FNA_P23 | 200 |  | 37310216 | 35875445 | 33277791 | 7.2 | 1272987312 | 38.3 | 1019.7 |
| FNA_P27 | 200 |  | 41029532 | 39143357 | 32146245 | 17.9 | 1208259466 | 37.6 | 968.2 |
| FNA_P28 | 200 |  | 38125438 | 36729515 | 30725383 | 16.4 | 1223398866 | 39.8 | 980.2 |
| FNA_P29 | 200 |  | 39563078 | 38166165 | 32272555 | 15.4 | 1272757171 | 39.4 | 1019.7 |
| FNA_P31 | 200 |  | 41606960 | 40160249 | 34275977 | 14.7 | 1354042309 | 39.5 | 1084.9 |
| FNA_P32 | 200 |  | 41176158 | 39721744 | 33329426 | 16.1 | 1343352504 | 40.3 | 1076.3 |
| FNA_P36 | 200 |  | 40517894 | 38781186 | 32465520 | 16.3 | 1231314725 | 37.9 | 986.6 |
| FNA_P37 | 200 |  | 38429720 | 37131495 | 30947249 | 16.7 | 1267610749 | 41.0 | 1015.8 |
| FNA_P42 | 200 |  | 34632222 | 33247744 | 28195424 | 15.2 | 1118053661 | 39.7 | 896.0 |
| FNA_P43 | 200 |  | 36273314 | 34754837 | 29405557 | 15.4 | 1189865804 | 40.5 | 953.6 |
| FNA_P46 | 200 |  | 40162530 | 38779832 | 32722182 | 15.6 | 1370884310 | 41.9 | 1098.3 |
| Plasma_P2.1 | 16.4 | 3 | 58908400 | 54159143 | 19579553 | 63.9 | 918410287 | 47.0 | 1960.9 |
| Plasma_P2.2 | 16.0 | 3 | 48421656 | 46366355 | 16469453 | 64.5 | 740754751 | 45.0 | 1581.6 |
| Plasma_P2.3 | 50.0 | 2 | 80962918 | 65453148 | 25229956 | 61.5 | 1362037725 | 54.0 | 2908.1 |
| Plasma_P5.1 | 32.6 | 2 | 56176830 | 45021792 | 21184094 | 53.0 | 1040189609 | 49.1 | 2220.9 |
| Plasma_P5.2 | 46.0 | 3 | 60885118 | 51453903 | 23873859 | 53.6 | 1352767378 | 56.7 | 2888.3 |
| Plasma_P5.3 | 50.0 | 2 | 79285894 | 67140222 | 27329884 | 59.3 | 1420387136 | 52.0 | 3032.7 |
| Plasma_P5.4 | 100.0 | 5 | 84179372 | 70129253 | 29743195 | 57.6 | 1553444844 | 52.2 | 3316.8 |
| Plasma_P5.5 | 81.5 | 5 | 85007956 | 68492243 | 28773759 | 58.0 | 1509220194 | 52.5 | 3222.3 |
| Plasma_P5.6 | 50.0 | 5 | 72313872 | 58781210 | 27228818 | 53.7 | 1437467087 | 52.8 | 3069.1 |
| Plasma_P5.7 | 212.0 | 5 | 144943310 | 120954281 | 55676021 | 54.0 | 2590996168 | 46.6 | 5532.0 |
| Plasma_P7.1 | 51.6 | 3 | 54146976 | 49667797 | 25230591 | 49.2 | 1308674430 | 51.9 | 2794.2 |
| Plasma_P7.2 | 50.0 | 5 | 50486016 | 47436032 | 24792038 | 47.7 | 1237458201 | 49.9 | 2642.1 |
| Plasma_P7.3 | 50.0 | 3 | 53817408 | 47527478 | 25043804 | 47.3 | 1321270034 | 52.8 | 2821.0 |
| Plasma_P7.4 | 46.4 | 2 | 7993412 | 6544342 | 4741376 | 27.6 | 287831363 | 60.7 | 614.5 |
| Plasma_P7.5 | 63.0 | 2 | 72704974 | 59321356 | 26471926 | 55.4 | 1339755097 | 50.6 | 2860.5 |
| Plasma_P7.6 | 15.8 | 5 | 82732466 | 66016188 | 24764332 | 62.5 | 1302355300 | 52.6 | 2780.7 |
| Plasma_P10.1 | 50.0 | 3 | 78115702 | 68882369 | 21620301 | 68.6 | 636984199 | 29.5 | 1360.0 |
| Plasma_P11.1 | 50.0 | 5 | 58420916 | 47733234 | 21926096 | 54.1 | 1200021429 | 54.8 | 2562.2 |
| Plasma_P11.2 | 37.0 | 3 | 49340010 | 40417382 | 18120374 | 55.2 | 991041644 | 54.7 | 2116.0 |
| Plasma_P11.3 | 15.8 | 5 | 48433600 | 41724760 | 15635564 | 62.5 | 730869606 | 46.8 | 1560.5 |
| Plasma_P11.4 | 28.8 | 4 | 62909042 | 57476499 | 23768537 | 58.7 | 1126354863 | 47.4 | 2404.9 |
| Plasma_P11.5 | 19.6 | 5 | 42756966 | 36043242 | 15283768 | 57.6 | 781237567 | 51.1 | 1668.0 |
| Plasma_P11.6 | 41.8 | 5 | 67735040 | 54606303 | 28561545 | 47.7 | 1267695457 | 44.4 | 2706.7 |
| Plasma_P21.1 | 31.8 | 2 | 70196552 | 56842930 | 23791666 | 58.1 | 1236256200 | 52.0 | 2639.5 |
| Plasma_P21.2 | 13.0 | 2 | 45642942 | 40857960 | 17386440 | 57.5 | 839911504 | 48.3 | 1793.3 |
| Plasma_P23.1 | 50.0 | 2 | 52943826 | 49582743 | 26478247 | 46.6 | 1288852648 | 48.7 | 2751.8 |
| Plasma_P23.2 | 16.2 | 2 | 28346322 | 23453597 | 10481901 | 55.3 | 521988433 | 49.8 | 1114.5 |
| Plasma_P23.3 | 50.0 | 2 | 60932214 | 49591829 | 23163803 | 53.3 | 1215541201 | 52.5 | 2595.3 |
| Plasma_P23.4 | 29.2 | 2 | 43291064 | 37281405 | 17234109 | 53.8 | 816575913 | 47.4 | 1743.5 |
| Plasma_P23.5 | 28.8 | 5 | 53614626 | 43684872 | 12754034 | 70.8 | 594432681 | 56.5 | 1269.2 |
| Plasma_P23.6 | 69.0 | 5 | 122884286 | 112298898 | 52476884 | 53.3 | 2083606132 | 39.8 | 4448.7 |
| Plasma_P23.7 | 102.0 | 5 | 71477508 | 66483585 | 39316623 | 40.9 | 1783356330 | 45.4 | 3807.6 |
| Plasma_P27.1 | 22.7 | 5 | 49417964 | 41249633 | 12176855 | 70.5 | 599503306 | 49.3 | 1280.0 |
| Plasma_P27.2 | 18.0 | 5 | 74098948 | 60879081 | 17884045 | 70.6 | 883381168 | 49.4 | 1886.1 |
| Plasma_P27.3 | 25.0 | 5 | 92338234 | 75130353 | 24816649 | 67.0 | 1272982724 | 51.3 | 2717.9 |
| Plasma_P27.4 | 21.5 | 5 | 169239066 | 137914265 | 37285229 | 73.0 | 1725399949 | 46.3 | 3683.9 |
| Plasma_P27.5 | 28.3 | 5 | 7281778 | 6173872 | 3836954 | 37.9 | 229624065 | 59.9 | 490.3 |
| Plasma_P27.6 | 15.3 | 5 | 41775952 | 37487946 | 16087932 | 57.1 | 787910567 | 49.0 | 1682.3 |
| Plasma_P27.7 | 15.5 | 5 | 67764950 | 63780104 | 24184580 | 62.1 | 938730999 | 38.8 | 2004.3 |
| Plasma_P27.8 | 7.8 | 5 | 32017772 | 26572355 | 11256275 | 57.6 | 503411014 | 44.7 | 1074.8 |
| Plasma_P28.1 | 10.2 | 3 | 59125234 | 51690870 | 15445880 | 70.1 | 634076221 | 41.1 | 1353.8 |
| Plasma_P28.2 | 26.6 | 3 | 77364536 | 69433217 | 24799551 | 64.3 | 1162827131 | 46.9 | 2482.8 |
| Plasma_P28.3 | 25.4 | 3 | 73867740 | 68534588 | 23573840 | 65.6 | 1045092738 | 44.4 | 2231.4 |
| Plasma_P28.4 | 22.7 | 3 | 66521474 | 62649863 | 22875243 | 63.5 | 1059364734 | 46.3 | 2261.9 |
| Plasma_P29.1 | 100.0 | 2 | 75733128 | 66726382 | 27116912 | 59.4 | 1437185292 | 53.0 | 3068.5 |
| Plasma_P29.2 | 81.5 | 2 | 57463410 | 47664041 | 17195611 | 63.9 | 903444515 | 52.6 | 1928.9 |
| Plasma_P29.3 | 63.0 | 2 | 82732466 | 66016188 | 24764332 | 62.5 | 1302355300 | 52.6 | 2780.7 |
| Plasma_P36.1 | 43.6 | 2 | 49638842 | 40976190 | 19810880 | 51.7 | 1058336616 | 53.4 | 2259.7 |
| Plasma_P36.2 | 49.3 | 2 | 52055110 | 41596138 | 20062718 | 51.8 | 1047420946 | 52.2 | 2236.3 |
| Plasma_P36.3 | 28.0 | 2 | 40137092 | 31765607 | 15047377 | 52.6 | 778910260 | 51.8 | 1663.1 |
| Plasma_P36.4 | 29.2 | 2 | 43707114 | 34847120 | 15659112 | 55.1 | 813037029 | 51.9 | 1735.9 |
| Plasma_P32.1 | 32.1 | 2 | 55499180 | 48211606 | 23813174 | 50.6 | 1213510487 | 51.0 | 2591.0 |
| Plasma_P31.1 | 10.2 | 2 | 53614626 | 43684872 | 12754034 | 70.8 | 594432681 | 46.6 | 1269.2 |
| Plasma_P31.2 | 26.6 | 2 | 41294592 | 33351074 | 9389496 | 71.9 | 442159607 | 47.1 | 944.1 |
| Plasma_P31.3 | 25.4 | 5 | 69500780 | 55702749 | 17721995 | 68.2 | 884027731 | 49.9 | 1887.5 |
| Plasma_P31.4 | 33.6 | 5 | 52776578 | 43195849 | 19677273 | 54.5 | 1015051735 | 51.6 | 2167.2 |
| Plasma_P37.1 | 25.5 | 3 | 52079076 | 47695120 | 19796710 | 58.5 | 942885042 | 47.6 | 2013.2 |
| Plasma_P37.2 | 30.3 | 3 | 58574650 | 51818528 | 24038976 | 53.6 | 1081781366 | 45.0 | 2309.7 |
| Plasma_P42.1 | 18.0 | 4 | 70052744 | 56555691 | 16632333 | 70.6 | 782212000 | 47.1 | 1670.1 |
| Plasma_P42.2 | 25.0 | 5 | 84547642 | 67458744 | 21965858 | 67.4 | 1089583625 | 49.6 | 2326.4 |
| Plasma_P42.3 | 21.5 | 5 | 83552566 | 66790192 | 21353948 | 68.0 | 1046370796 | 49.0 | 2234.1 |
| Plasma_P42.4 | 28.3 | 5 | 19726608 | 16225205 | 7827663 | 51.8 | 448225291 | 57.3 | 957.0 |
| Plasma_P42.5 | 18.3 | 5 | 97770534 | 76225669 | 28562409 | 62.5 | 1087040537 | 38.2 | 2320.9 |
| Plasma_P43.1 | 10.3 | 3 | 44397120 | 40079168 | 14675984 | 63.4 | 660638307 | 45.0 | 1410.5 |
| Plasma_P43.2 | 14.9 | 5 | 57033178 | 45774843 | 17491337 | 61.8 | 863302113 | 49.4 | 1843.2 |
| Plasma_P43.3 | 21.3 | 5 | 44084976 | 35424665 | 15217749 | 57.0 | 795761114 | 52.3 | 1699.0 |
| Plasma_P43.4 | 34.4 | 5 | 108203116 | 86235890 | 33282590 | 61.4 | 1413965720 | 42.5 | 3019.0 |
| Plasma_P43.5 | 26.9 | 5 | 47317632 | 37854103 | 20181271 | 46.7 | 957449259 | 47.5 | 2044.3 |
| Plasma_P46.1 | 15.2 | 2 | 42262700 | 39004499 | 14445711 | 63.0 | 638873433 | 44.2 | 1364.1 |
| PBL_P2.1 | 200 |  | 21205080 | 19957102 | 16428442 | 17.7 | 837358771 | 51.0 | 1787.8 |
| PBL_P5.1_A | 200 |  | 19744110 | 18518807 | 15136631 | 18.3 | 814943029 | 53.9 | 1740.0 |
| PBL_P5.1_B | 200 |  | 30991224 | 28675217 | 23397333 | 18.4 | 874698305 | 37.4 | 1867.6 |
| PBL_P5.2 | 200 |  | 33061534 | 30672861 | 25009511 | 18.5 | 920927696 | 36.8 | 1966.3 |
| PBL_P5.6 | 200 |  | 27697486 | 25737450 | 21318442 | 17.2 | 790717636 | 37.1 | 1688.3 |
| PBL_P5.7 | 200 |  | 31267838 | 28916069 | 22842185 | 21.0 | 812883497 | 35.6 | 1735.6 |
| PBL_P7.1 | 200 |  | 21951602 | 20597201 | 16481247 | 20.0 | 883504133 | 53.6 | 1886.4 |
| PBL_P10.1 | 200 |  | 14487458 | 13673410 | 9584416 | 29.9 | 488091075 | 50.9 | 1042.1 |
| PBL_P11.1 | 200 |  | 18515010 | 17377331 | 12599633 | 27.5 | 649530947 | 51.6 | 1386.8 |
| PBL_P11.6 | 200 |  | 31070996 | 29557448 | 22597142 | 23.6 | 1331777657 | 59.0 | 2843.5 |
| PBL_P21.1 | 200 |  | 21454038 | 20123870 | 17306910 | 14.0 | 951356634 | 55.0 | 2031.2 |
| PBL_P23.1 | 200 |  | 18961016 | 17757499 | 12814381 | 27.8 | 664522008 | 51.9 | 1418.8 |
| PBL_P23.6 | 200 |  | 122884286 | 112298898 | 52476884 | 53.3 | 1150953331 | 59.5 | 2457.4 |
| PBL_P23.7 | 200 |  | 26510484 | 24948747 | 19768785 | 20.8 | 1100235471 | 55.7 | 2349.1 |
| PBL_P27.1_A | 200 |  | 17152872 | 16273964 | 13444864 | 17.4 | 730843371 | 54.4 | 1560.4 |
| PBL_P27.1_B | 200 |  | 24261482 | 22532691 | 18100193 | 19.7 | 673073494 | 37.2 | 1437.1 |
| PBL_P27.2 | 200 |  | 27284136 | 25426331 | 21126923 | 16.9 | 786431455 | 37.2 | 1679.1 |
| PBL_P27.6 | 200 |  | 28159468 | 26391474 | 20401062 | 22.7 | 966863179 | 47.4 | 2064.4 |
| PBL_P27.7 | 200 |  | 26765972 | 25135790 | 19128146 | 23.9 | 919871366 | 48.1 | 1964.0 |
| PBL_P27.8 | 200 |  | 26818554 | 25417921 | 19814401 | 22.1 | 1172197768 | 59.2 | 2502.8 |
| PBL_P28.1 | 200 |  | 19654052 | 18421274 | 15178144 | 17.6 | 829902469 | 54.7 | 1771.9 |
| PBL_P29.1 | 200 |  | 16204016 | 15153788 | 10740150 | 29.1 | 550413845 | 51.3 | 1175.2 |
| PBL_P31.1 | 200 |  | 25649200 | 23929900 | 17425102 | 27.2 | 952482757 | 54.7 | 2033.6 |
| PBL_P32.1 | 200 |  | 22966004 | 21575833 | 18522475 | 14.2 | 1017656032 | 55.0 | 2172.8 |
| PBL_P36.1 | 200 |  | 21708366 | 20093522 | 16203606 | 19.4 | 881725246 | 54.4 | 1882.6 |
| PBL_P36.4 | 200 |  | 25280774 | 23836805 | 18762979 | 21.3 | 1089330548 | 58.1 | 2325.8 |
| PBL_P37.1 | 200 |  | 25061002 | 23597521 | 19994639 | 15.3 | 1105955712 | 55.3 | 2361.3 |
| PBL_P42.1 | 200 |  | 18603962 | 17393922 | 12862838 | 26.1 | 689759809 | 53.6 | 1472.7 |
| PBL_P43.1_A | 200 |  | 24224658 | 22701496 | 18971612 | 16.4 | 1044144989 | 55.1 | 2229.4 |
| PBL_P43.1_B | 200 |  | 24829080 | 23554408 | 18773820 | 20.3 | 1112967016 | 59.3 | 2376.3 |
| PBL_P43.2 | 200 |  | 31187754 | 29275809 | 22137921 | 24.4 | 1062689793 | 48.0 | 2268.9 |
| PBL_P43.3 | 200 |  | 27700874 | 25763434 | 19728904 | 23.4 | 931406357 | 47.3 | 1988.6 |
| PBL_P43.5 | 200 |  | 26229890 | 24462721 | 18894377 | 22.8 | 917459901 | 48.6 | 1958.9 |
| PBL_P46.1 | 200 |  | 22807320 | 21353272 | 18201380 | 14.8 | 1004092084 | 55.2 | 2143.8 |

| **Supplementary Table S2. The list of genes selected for targeted deep sequencing.** | | | | | | | |
| --- | --- | --- | --- | --- | --- | --- | --- |
| ABL1 | AURKA | CTNNB1 | FGFR2 | ITK | MTOR | PTCH2 | SYK |
| AKT1 | AURKB | DDR2 | FGFR3 | JAK1 | NF1 | PTEN | TERT |
| AKT2 | BCL2 | EGFR | FLT3 | JAK2 | NOTCH1 | PTPN11 | TOP1 |
| AKT3 | BRAF | EPHB4 | GNA11 | JAK3 | NPM1 | RB1 | TP53 |
| ALK | BRCA1 | ERBB2 | GNAQ | KDR | NRAS | RET | TMPRSS2 |
| APC | BRCA2 | ERBB3 | GNAS | KIT | NTRK1 | ROS1 | VHL |
| ARID1A | CDH1 | ERBB4 | HNF1A | KRAS | PDGFRA | SMAD4 |  |
| ARID1B | CDK4 | EWSR1 | HRAS | MDM2 | PDGFRB | SMARCB1 | |
| ARID2 | CDK6 | EZH2 | IDH1 | MET | PIK3CA | SMO |  |
| ATM | CDKN2A | FBXW7 | IDH2 | MLH1 | PIK3R1 | SRC |  |
| ATRX | CSF1R | FGFR1 | IGF1R | MPL | PTCH1 | STK11 |  |

| **Supplementary Table S3. Detection of ctDNA using droplet digital PCR.** | | | | | |  |
| --- | --- | --- | --- | --- | --- | --- |
| **Sample ID** | **Gene** | **AA Change** | **Input DNA (ng)** | **Mutant (copies/mL)** | **Wildtype (copies/mL)** | **MAF (%)** |
| P2.1 | KRAS | G12D | 5.55 | 0.0 | 3062.5 | 0.00 |
| P2.1 | KRAS | G12V | 5.55 | 0.0 | 2951.7 | 0.00 |
| P2.1 | ROS1 | I1967V | 5.55 | 180.8 | 3710.0 | 4.87 |
| P2.2 | KRAS | G12D | 3.60 | 5.3 | 4433.3 | 0.12 |
| P2.3 | KRAS | G12D | 8.80 | 140.0 | 23887.5 | 0.59 |
| P5.1 | KRAS | G12D | 8.32 | 0.0 | 10587.5 | 0.00 |
| P5.1 | KRAS | G12V | 8.32 | 0.0 | 10587.5 | 0.00 |
| P5.1 | RB1 | R251* | 8.32 | 16.6 | 23100.0 | 0.07 |
| P5.2 | RB1 | R251* | 11.12 | 58.3 | 13883.3 | 0.42 |
| P5.3 | RB1 | R251* | 21.60 | 7.0 | 35350.0 | 0.02 |
| P5.4 | RB1 | R251* | 41.92 | 5.6 | 35000.0 | 0.02 |
| P5.5 | RB1 | R251* | 11.92 | 273.0 | 10780.0 | 2.53 |
| P5.6 | RB1 | R251* | 13.44 | 952.0 | 7700.0 | 12.36 |
| P5.7 | RB1 | R251* | 106.00 | 24850.0 | 33880.0 | 73.35 |
| P7.1 | KRAS | G12V | 10.32 | 0.0 | 8575.0 | 0.00 |
| P7.2 | KRAS | G12V | 12.80 | 0.0 | 6020.0 | 0.00 |
| P7.3 | KRAS | G12V | 15.68 | 0.0 | 15166.7 | 0.00 |
| P7.4 | KRAS | G12V | 9.52 | 0.0 | 12950.0 | 0.00 |
| P7.5 | KRAS | G12V | 6.27 | 0.0 | 6693.8 | 0.00 |
| P7.5 | KRAS | G12D | 6.27 | 0.0 | 6746.3 | 0.00 |
| P7.6 | KRAS | G12V | 26.72 | 31.5 | 5250.0 | 0.60 |
| P10.1 | KRAS | G12D | 9.20 | 180.8 | 7058.3 | 2.56 |
| P10.1 | KRAS | G12V | 9.20 | 0.0 | 8575.0 | 0.00 |
| P21.1 | KRAS | G12V | 8.48 | 218.8 | 10587.5 | 2.07 |
| P21.2 | KRAS | G12V | 2.82 | 105.0 | 4716.3 | 2.23 |
| P23.1 | KRAS | G12V | 2.32 | 41.1 | 22750.0 | 0.18 |
| P23.1 | KRAS | G12D | 2.32 | 0.0 | 2336.3 | 0.00 |
| P23.2 | KRAS | G12V | 3.17 | 0.0 | 113.8 | 0.00 |
| P23.2 | KRAS | G12D | 3.17 | 0.0 | 1732.5 | 0.00 |
| P23.3 | KRAS | G12V | 7.76 | 0.0 | 12250.0 | 0.00 |
| P23.4 | KRAS | G12V | 2.59 | 0.0 | 3788.8 | 0.00 |
| P23.4 | KRAS | G12D | 1.82 | 0.0 | 1675.0 | 0.00 |
| P23.5 | KRAS | G12V | 28.64 | 0.0 | 7175.0 | 0.00 |
| P23.6 | KRAS | G12V | 11.04 | 0.0 | 8120.0 | 0.00 |
| P23.7 | KRAS | G12V | 102.00 | 0.0 | 9135.0 | 0.00 |
| P27.1 | KRAS | G12D | 1.25 | 0.0 | 675.5 | 0.00 |
| P27.1 | KRAS | G12V | 1.25 | 0.0 | 546.0 | 0.00 |
| P27.2 | KRAS | G12D | 4.22 | 0.0 | 1379.0 | 0.00 |
| P27.2 | KRAS | G12V | 3.18 | 0.0 | 1281.0 | 0.00 |
| P27.3 | KRAS | G12D | 9.20 | 0.0 | 3780.0 | 0.00 |
| P27.4 | KRAS | G12D | 8.32 | 0.0 | 3710.0 | 0.00 |
| P27.5 | KRAS | G12D | 4.99 | 0.0 | 1869.0 | 0.00 |
| P27.6 | KRAS | G12D | 9.76 | 0.0 | 1246.0 | 0.00 |
| P27.6 | KRAS | G12V | 2.37 | 0.0 | 962.5 | 0.00 |
| P27.7 | KRAS | G12D | 2.48 | 0.0 | 1106.0 | 0.00 |
| P27.8 | KRAS | G12D | 1.25 | 0.0 | 546.0 | 0.00 |
| P29.1 | KRAS | G12V | 4.19 | 113.8 | 3491.3 | 3.26 |
| P29.2 | KRAS | G12V | 7.22 | 44.6 | 7017.5 | 0.64 |
| P29.3 | KRAS | G12V | 3.44 | 70.0 | 2310.0 | 3.03 |
| P31.1 | KRAS | G12D | 11.12 | 113.8 | 1408.8 | 8.07 |
| P31.2 | KRAS | G12D | 26.24 | 0.0 | 7262.5 | 0.00 |
| P31.3 | KRAS | G12D | 30.88 | 0.0 | 5022.5 | 0.00 |
| P31.4 | KRAS | G12D | 4.11 | 0.0 | 4506.3 | 0.00 |
| P32.1 | KRAS | G12D | 6.00 | 122.5 | 5766.3 | 2.12 |
| P32.1 | KRAS | G12V | 6.00 | 5.3 | 5110.0 | 0.10 |
| P36.1 | KRAS | G12D | 3.28 | 183.8 | 10587.5 | 1.74 |
| P36.2 | KRAS | G12D | 1.86 | 57.8 | 7262.5 | 0.80 |
| P36.3 | KRAS | G12D | 15.84 | 18.6 | 2002.0 | 0.93 |
| P36.4 | KRAS | G12D | 11.44 | 49.0 | 1795.5 | 2.73 |
| P37.1 | KRAS | G12D | 5.23 | 134.2 | 3045.0 | 4.41 |
| P37.1 | KRAS | G12V | 5.23 | 0.0 | 3126.7 | 0.00 |
| P37.2 | KRAS | G12D | 6.22 | 49.0 | 3424.2 | 1.43 |
| P43.1 | KRAS | G12D | 2.66 | 4.7 | 2677.5 | 0.17 |
| P43.2 | KRAS | G12D | 2.45 | 2.1 | 1064.0 | 0.20 |
| P43.2 | KRAS | G12V | 3.54 | 0.0 | 1029.0 | 0.00 |
| P43.3 | KRAS | G12D | 2.86 | 0.0 | 1302.0 | 0.00 |
| P43.3 | KRAS | G12V | 4.26 | 0.0 | 1060.5 | 0.00 |
| P43.4 | KRAS | G12D | 5.50 | 28.0 | 2093.0 | 1.34 |
| P46.1 | KRAS | G12D | 2.54 | 6.1 | 1820.0 | 0.34 |
| P46.1 | KRAS | G12V | 2.54 | 0.0 | 2773.8 | 0.00 |
| AA, amino acid; MAF, mutant allele frequency. | | | |  |  |  |

**Supplementary Table S4. Comparison of KRAS mutation detection between droplet digital PCR and cfDNA sequencing.**

| **Sample ID** | **Gene** | **AA Change** | **ddPCR** | **NGS** |
| --- | --- | --- | --- | --- |
| P2.1 | KRAS | G12D | - | - |
| P2.1 | KRAS | G12V | - | - |
| P2.2 | KRAS | G12D | + | + |
| P2.3 | KRAS | G12D | + | + |
| P5.1 | KRAS | G12D | - | - |
| P5.1 | KRAS | G12V | - | - |
| P7.1 | KRAS | G12V | - | - |
| P7.2 | KRAS | G12V | - | - |
| P7.3 | KRAS | G12V | - | - |
| P7.4 | KRAS | G12V | - | - |
| P7.5 | KRAS | G12V | - | - |
| P7.5 | KRAS | G12D | - | - |
| P7.6 | KRAS | G12V | - | - |
| P10.1 | KRAS | G12D | + | + |
| P10.1 | KRAS | G12V | - | - |
| P21.1 | KRAS | G12V | + | + |
| P21.2 | KRAS | G12V | + | + |
| P23.1 | KRAS | G12V | + | + |
| P23.1 | KRAS | G12D | - | - |
| P23.2 | KRAS | G12V | - | - |
| P23.2 | KRAS | G12D | - | - |
| P23.3 | KRAS | G12V | - | - |
| P23.4 | KRAS | G12V | - | - |
| P23.4 | KRAS | G12D | - | - |
| P23.5 | KRAS | G12V | - | - |
| P23.6 | KRAS | G12V | - | - |
| P23.7 | KRAS | G12V | - | - |
| P27.1 | KRAS | G12D | - | - |
| P27.1 | KRAS | G12V | - | - |
| P27.2 | KRAS | G12D | - | - |
| P27.2 | KRAS | G12V | - | - |
| P27.3 | KRAS | G12D | - | - |
| P27.4 | KRAS | G12D | - | - |
| P27.5 | KRAS | G12D | - | - |
| P27.6 | KRAS | G12D | - | - |
| P27.6 | KRAS | G12V | - | - |
| P27.7 | KRAS | G12D | - | - |
| P27.8 | KRAS | G12D | - | - |
| P29.1 | KRAS | G12V | + | + |
| P29.2 | KRAS | G12V | + | + |
| P29.3 | KRAS | G12V | + | + |
| P31.1 | KRAS | G12D | + | + |
| P31.2 | KRAS | G12D | - | - |
| P31.3 | KRAS | G12D | - | - |
| P31.4 | KRAS | G12D | - | - |
| P32.1 | KRAS | G12D | + | + |
| P32.1 | KRAS | G12V | + | - |
| P36.1 | KRAS | G12D | + | + |
| P36.2 | KRAS | G12D | + | + |
| P36.3 | KRAS | G12D | + | + |
| P36.4 | KRAS | G12D | + | + |
| P37.1 | KRAS | G12D | + | + |
| P37.1 | KRAS | G12V | - | - |
| P37.2 | KRAS | G12D | + | + |
| P43.1 | KRAS | G12D | + | + |
| P43.2 | KRAS | G12D | + | + |
| P43.2 | KRAS | G12V | - | - |
| P43.3 | KRAS | G12D | - | - |
| P43.3 | KRAS | G12V | - | - |
| P43.4 | KRAS | G12D | + | + |
| P46.1 | KRAS | G12D | + | + |
| P46.1 | KRAS | G12V | - | - |
| AA, amino acid; ddPCR, droplet digital PCR. | | | | |

**Supplementary Table S5. The list of somatic mutations obtained from fine needle aspiration.**

| **Sample ID** | **Gene** | **AA or NT change** | **RefSeq ID** | **Chr** | **Position** | **Ref** | **Alt** | **Mutation class** | **Primary VAF (%)** | **Mutect/Varscan** |
| --- | --- | --- | --- | --- | --- | --- | --- | --- | --- | --- |
| FNA_P2 | KRAS | p.G12D | NM_033360 | chr12 | 25398284 | C | T | Missense | 21.1 | Mutect/Varscan |
| FNA_P2 | TP53 | p.Y236C | NM_001126113 | chr17 | 7577574 | T | C | Missense | 25.4 | Mutect/Varscan |
| FNA_P5 | MET | p.S889F | NM_000245 | chr7 | 116409781 | C | T | Missense | 33.6 | Mutect/Varscan |
| FNA_P5 | SMAD4 | p.Q256X | NM_005359 | chr18 | 48584593 | C | T | Nonsense | 9.3 | Mutect/Varscan |
| FNA_P5 | TP53 | c.96+1G>A | NM_001126113 | chr17 | 7579699 | C | T | Splicing variant | 65.6 | Varscan |
| FNA_P7 | KRAS | p.G12V | NM_033360 | chr12 | 25398284 | C | A | Missense | 8.7 | Varscan |
| FNA_P7 | TP53 | p.R175H | NM_001126113 | chr17 | 7578406 | C | T | Missense | 8.5 | Varscan |
| FNA_P10 | KRAS | p.G12D | NM_033360 | chr12 | 25398284 | C | T | Missense | 13.9 | Mutect/Varscan |
| FNA_P10 | TP53 | c.386+1G>A | NM_001126113 | chr17 | 7577498 | C | T | Splicing variant | 14.9 | Varscan |
| FNA_P21 | KRAS | p.G12V | NM_033360 | chr12 | 25398284 | C | A | Missense | 14.7 | Mutect/Varscan |
| FNA_P21 | TP53 | p.I30S | NM_001126113 | chr17 | 7578445 | A | C | Missense | 20.8 | Varscan |
| FNA_P23 | KRAS | p.G12V | NM_033360 | chr12 | 25398284 | C | A | Missense | 16.4 | Mutect/Varscan |
| FNA_P23 | SMAD4 | p.R361C | NM_005359 | chr18 | 48591918 | C | T | Missense | 23.3 | Mutect/Varscan |
| FNA_P27 | KRAS | p.G12D | NM_033360 | chr12 | 25398284 | C | T | Missense | 3.6 | Mutect |
| FNA_P29 | KRAS | p.G12V | NM_033360 | chr12 | 25398284 | C | A | Missense | 4.5 | Mutect/Varscan |
| FNA_P29 | ARID1B | p.Q2092X | NM_020732 | chr6 | 157528549 | C | T | Nonsense | 3.8 | Mutect |
| FNA_P29 | EGFR | p.K189E | NM_005228 | chr7 | 55218992 | A | G | Missense | 4.7 | Mutect |
| FNA_P29 | ERBB3 | p.P583S | NM_001982 | chr12 | 56488228 | C | T | Missense | 4.2 | Mutect/Varscan |
| FNA_P29 | TP53 | p.L194R | NM_001126113 | chr17 | 7578268 | A | C | Missense | 4.8 | Mutect |
| FNA_P31 | KRAS | p.G12D | NM_033360 | chr12 | 25398284 | C | T | Missense | 38.6 | Mutect/Varscan |
| FNA_P31 | ATRX | p.A3T | NM_000489 | chrX | 77041481 | C | T | Missense | 6.1 | Varscan |
| FNA_P31 | TP53 | p.F113C | NM_001126113 | chr17 | 7579349 | A | C | Missense | 54.5 | Mutect/Varscan |
| FNA_P32 | KRAS | p.G12D | NM_033360 | chr12 | 25398284 | C | T | Missense | 19.7 | Mutect/Varscan |
| FNA_P32 | TP53 | p.H154R | NM_001126113 | chr17 | 7578271 | T | C | Missense | 28.0 | Mutect/Varscan |
| FNA_P36 | KRAS | p.G12D | NM_033360 | chr12 | 25398284 | C | T | Missense | 16.9 | Mutect/Varscan |
| FNA_P36 | TP53 | p.Y220C | NM_001126113 | chr17 | 7578190 | T | C | Missense | 23.7 | Mutect/Varscan |
| FNA_P37 | CDKN2A | p.C100X | NM_058195 | chr9 | 21971101 | G | T | Nonsense | 61.0 | Mutect/Varscan |
| FNA_P37 | KRAS | p.G12D | NM_033360 | chr12 | 25398284 | C | T | Missense | 55.6 | Varscan |
| FNA_P37 | TP53 | p.R119L | NM_001126113 | chr17 | 7578457 | C | A | Missense | 60.0 | Mutect |
| FNA_P42 | TP53 | p.L226P | NM_001126113 | chr17 | 7577144 | A | G | Missense | 3.4 | Mutect |
| FNA_P42 | SMAD4 | p.R361C | NM_005359 | chr18 | 48591918 | C | T | Missense | 4.5 | Varscan |
| FNA_P42 | EPHB4 | p.F404L | NM_004444 | chr7 | 100417264 | A | C | Missense | 9.3 | Mutect/Varscan |
| FNA_P43 | KRAS | p.G12D | NM_033360 | chr12 | 25398284 | C | T | Missense | 28.6 | Varscan |
| FNA_P43 | CDKN2A | p.A97V | NM_058195 | chr9 | 21971111 | G | A | Missense | 23.1 | Varscan |
| FNA_P43 | TP53 | p.D259V | NM_001126113 | chr17 | 7577505 | T | A | Missense | 29.6 | Mutect/Varscan |
| FNA_P46 | PDGFRB | p.P866S | NM_002609 | chr5 | 149499677 | G | A | Missense | 17.1 | Mutect |
| FNA_P46 | PTCH1 | c.3606+1G>A | NM_001083602 | chr9 | 98211350 | C | T | Splicing variant | 21.7 | Mutect/Varscan |
| FNA_P46 | KRAS | p.G12D | NM_033360 | chr12 | 25398284 | C | T | Missense | 33.1 | Varscan |
| FNA_P46 | PDGFRB | p.L865F | NM_002609 | chr5 | 149499678 | C | G | Missense | 18.3 | Varscan |
| FNA_P46 | TP53 | p.Q167X | NM_001126113 | chr17 | 7578431 | G | A | Nonsense | 25.1 | Mutect/Varscan |
| AA, amino acid; NT, nucleotide; VAF, variant allele frequency. | | | | |  |  |  |  |  |  |

**Supplementary Table S6. Somatic mutations detected in 17 patient plasma samples.**

| **Sample ID** | **Chr** | **Position** | **Ref** | **Alt** | **Gene** | **Mutation class** | **AA or NT Change** | **RefSeq ID** | **Read Count** | **Total read** | **Allele frequency** | **M_P/FNA_ *p*-value** | **M_P/TR_ *p*-value** |
| --- | --- | --- | --- | --- | --- | --- | --- | --- | --- | --- | --- | --- | --- |
| P2.1 | chr6 | 117641072 | T | C | ROS1 | Missense | p.I1967V | NM_002944 | 1252 | 3246 | 38.57 | ND | 0.E+00 |
| P2.3 | chr12 | 25398284 | C | T | KRAS | Missense | p.G12D | NM_033360 | 6 | 3198 | 0.19 | 7.E-04 | NS |
| P2.3 | chr17 | 7577574 | T | C | TP53 | Missense | p.Y236C | NM_001126113 | 9 | 2517 | 0.36 | 3.E-04 | NS |
| P5.1 | chr6 | 117609741 | G | A | ROS1 | Nonsense | p.Q2320X | NM_002944 | 20 | 2696 | 0.74 | ND | 4.E-21 |
| P5.1 | chr8 | 38277157 | G | A | FGFR1 | Missense | p.S385L | NM_001174064 | 20 | 1494 | 1.34 | ND | 3.E-26 |
| P5.5 | chr7 | 116409781 | C | T | MET | Missense | p.S889F | NM_000245 | 62 | 3057 | 2.03 | 2.E-04 | 2.E-96 |
| P5.5 | chr13 | 48919281 | C | G | RB1 | Nonsense | p.S149X | NM_000321 | 56 | 3624 | 1.55 | ND | 2.E-80 |
| P5.5 | chr13 | 48936983 | C | T | RB1 | Nonsense | p.R251X | NM_000321 | 105 | 3357 | 3.13 | ND | 2.E-184 |
| P5.5 | chr17 | 7579699 | C | T | TP53 | Splicing variant | c.96+1G>A | NM_001126113 | 69 | 2246 | 3.07 | 2.E-04 | 4.E-120 |
| P5.6 | chr7 | 116409781 | C | T | MET | Missense | p.S889F | NM_000245 | 275 | 2781 | 9.89 | 2.E-04 | 0.E+00 |
| P5.6 | chr13 | 48919281 | C | G | RB1 | Nonsense | p.S149X | NM_000321 | 300 | 3188 | 9.41 | ND | 0.E+00 |
| P5.6 | chr13 | 48936983 | C | T | RB1 | Nonsense | p.R251X | NM_000321 | 358 | 3447 | 10.39 | ND | 0.E+00 |
| P5.6 | chr17 | 7579699 | C | T | TP53 | Splicing variant | c.96+1G>A | NM_001126113 | 311 | 1928 | 16.13 | 2.E-04 | 0.E+00 |
| P5.7 | chr7 | 116409781 | C | T | MET | Missense | p.S889F | NM_000245 | 2057 | 4675 | 44.00 | 2.E-04 | 0.E+00 |
| P5.7 | chr13 | 48919281 | C | G | RB1 | Nonsense | p.S149X | NM_000321 | 2191 | 5092 | 43.03 | ND | 0.E+00 |
| P5.7 | chr13 | 48936983 | C | T | RB1 | Nonsense | p.R251X | NM_000321 | 2677 | 5907 | 45.32 | ND | 0.E+00 |
| P5.7 | chr17 | 7579472 | G | C | TP53 | Missense | p.P72R | NM_001126113 | 34 | 3128 | 1.09 | ND | 6.E-43 |
| P5.7 | chr17 | 7579699 | C | T | TP53 | Splicing variant | c.96+1G>A | NM_001126113 | 2313 | 2696 | 85.79 | 9.E-05 | 0.E+00 |
| P5.7 | chrX | 76855029 | T | C | ATRX | Missense | p.K1936R | NM_000489 | 33 | 6527 | 0.51 | ND | 7.E-31 |
| P5.1 | chr7 | 116409781 | C | T | MET | Missense | p.S907F | NM_000245 | 7 | 2295 | 0.31 | 3.E-04 | NS |
| P5.2 | chr7 | 116409781 | C | T | MET | Missense | p.S907F | NM_000245 | 7 | 2699 | 0.26 | 3.E-04 | NS |
| P5.2 | chr17 | 7579699 | C | T | TP53 | Splicing variant | c.96+1G>A | NM_001126113 | 7 | 2314 | 0.30 | 3.E-04 | NS |
| P7.5 | chr17 | 7578406 | C | T | TP53 | Missense | p.R175H | NM_001126113 | 9 | 1979 | 0.45 | 3.E-04 | NS |
| P7.6 | chr12 | 25398284 | C | A | KRAS | Missense | p.G12V | NM_033360 | 16 | 3134 | 0.51 | 2.E-04 | NS |
| P7.1 | chr6 | 117710794 | C | T | ROS1 | Missense | p.R493H | NM_002944 | 29 | 2974 | 0.98 | ND | 5.E-35 |
| P7.2 | chr6 | 117710794 | C | T | ROS1 | Missense | p.R493H | NM_002944 | 40 | 2806 | 1.43 | ND | 1.E-55 |
| P7.3 | chr6 | 117710794 | C | T | ROS1 | Missense | p.R493H | NM_002944 | 34 | 2770 | 1.23 | ND | 1.E-44 |
| P7.5 | chr6 | 117710794 | C | T | ROS1 | Missense | p.R493H | NM_002944 | 45 | 3031 | 1.48 | ND | 2.E-63 |
| P7.6 | chr6 | 117710794 | C | T | ROS1 | Missense | p.R493H | NM_002944 | 40 | 3137 | 1.28 | ND | 1.E-53 |
| P10.1 | chr12 | 25398284 | C | T | KRAS | Missense | p.G12D | NM_033360 | 30 | 1367 | 2.19 | 2.E-04 | 4.E-46 |
| P10.1 | chr17 | 7577498 | C | T | TP53 | Splicing variant | c.782+1G>A | NM_001126113 | 48 | 1167 | 4.11 | 2.E-04 | 2.E-88 |
| P11.5 | chr17 | 7574021 | C | A | TP53 | Nonsense | p.E297X | NM_001276761 | 28 | 1742 | 1.61 | ND | 1.E-39 |
| P21.1 | chr17 | 7578445 | A | C | TP53 | Missense | p.I162S | NM_001126113 | 41 | 2410 | 1.70 | 2.E-04 | 3.E-60 |
| P21.2 | chr9 | 133759935 | G | T | ABL1 | Missense | p.G772V | NM_007313 | 17 | 1891 | 0.90 | ND | 4.E-19 |
| P21.2 | chr10 | 43610119 | G | A | RET | Missense | p.G691S | NM_020975 | 29 | 1141 | 2.54 | ND | 6.E-47 |
| P21.2 | chr12 | 46246206 | G | T | ARID2 | Missense | p.A1434S | NM_152641 | 28 | 2165 | 1.29 | ND | 4.E-37 |
| P21.2 | chr16 | 68857389 | A | G | CDH1 | Missense | p.K675R | NM_004360 | 38 | 1943 | 1.96 | ND | 8.E-58 |
| P21.2 | chrX | 76938923 | G | C | ATRX | Missense | p.P609A | NM_000489 | 30 | 1065 | 2.82 | ND | 5.E-50 |
| P21.1 | chr12 | 25398284 | C | A | KRAS | Missense | p.G12V | NM_033360 | 36 | 2935 | 1.23 | 2.E-04 | NS |
| P21.2 | chr12 | 25398284 | C | A | KRAS | Missense | p.G12V | NM_033360 | 4 | 1736 | 0.23 | 1.E-03 | NS |
| P21.2 | chr17 | 7578445 | A | C | TP53 | Missense | p.I162S | NM_001126113 | 16 | 1894 | 0.84 | 3.E-04 | NS |
| P23.1 | chr7 | 148512600 | T | C | EZH2 | Missense | p.K515R | NM_004456 | 19 | 2243 | 0.85 | ND | 4.E-21 |
| P23.6 | chr6 | 117642495 | C | T | ROS1 | Missense | p.E1902K | NM_002944 | 19 | 2065 | 0.92 | ND | 1.E-21 |
| P23.6 | chrX | 76938208 | A | G | ATRX | Missense | p.F847S | NM_000489 | 24 | 3887 | 0.62 | ND | 9.E-24 |
| P23.1 | chr12 | 25398284 | C | A | KRAS | Missense | p.G12V | NM_033360 | 5 | 2909 | 0.17 | 7.E-04 | NS |
| P27.3 | chr1 | 27102188 | A | G | ARID1A | Missense | p.N1705S | NM_006015 | 35 | 3306 | 1.06 | ND | 5.E-44 |
| P27.3 | chr3 | 178927410 | A | G | PIK3CA | Missense | p.I391M | NM_006218 | 19 | 2986 | 0.64 | ND | 6.E-19 |
| P27.3 | chr11 | 108106443 | T | A | ATM | Missense | p.D126E | NM_000051 | 23 | 2498 | 0.92 | ND | 6.E-27 |
| P27.4 | chr2 | 29917793 | C | T | ALK | Missense | p.R292H | NM_004304 | 21 | 3837 | 0.55 | ND | 9.E-20 |
| P28.1 | chr10 | 89720875 | G | T | PTEN | Missense | p.K342N | NM_000314 | 39 | 998 | 3.91 | ND | 1.E-70 |
| P28.2 | chr9 | 98224138 | C | A | PTCH1 | Missense | p.Q835H | NM_001083602 | 383 | 2835 | 13.51 | ND | 0.E+00 |
| P28.2 | chr10 | 89720875 | G | T | PTEN | Missense | p.K342N | NM_000314 | 34 | 4033 | 0.84 | ND | 3.E-39 |
| P29.1 | chr7 | 55218992 | A | G | EGFR | Missense | p.K189E | NM_005228 | 28 | 2268 | 1.23 | 2.E-04 | 1.E-36 |
| P29.1 | chr6 | 157528549 | C | T | ARID1B | Missense | p.Q2092X | NM_020732 | 8 | 2495 | 0.32 | 3.E-04 | NS |
| P29.1 | chr7 | 55218992 | A | G | ERBB3 | Missense | p.P583S | NM_005228 | 11 | 2340 | 0.47 | 2.E-04 | NS |
| P29.1 | chr17 | 7578268 | A | C | TP53 | Missense | p.L194R | NM_001126113 | 8 | 1906 | 0.42 | 2.E-04 | NS |
| P29.1 | chr12 | 25398284 | C | A | KRAS | Missense | p.G12V | NM_033360 | 9 | 2267 | 0.40 | 3.E-04 | NS |
| P29.2 | chr12 | 25398284 | C | A | KRAS | Missense | p.G12V | NM_033360 | 7 | 3190 | 0.22 | 4.E-04 | NS |
| P29.3 | chr6 | 157528549 | C | T | ARID1B | Missense | p.Q2092X | NM_020732 | 6 | 2269 | 0.26 | 5.E-04 | NS |
| P29.3 | chr7 | 55218992 | A | G | EGFR | Missense | p.K189E | NM_005228 | 11 | 2253 | 0.49 | 2.E-04 | NS |
| P29.3 | chr7 | 55218992 | A | G | ERBB3 | Missense | p.P583S | NM_005228 | 13 | 2300 | 0.57 | 2.E-04 | NS |
| P29.3 | chr12 | 25398284 | C | A | KRAS | Missense | p.G12V | NM_033360 | 11 | 2124 | 0.52 | 2.E-04 | NS |
| P31.1 | chr12 | 25398284 | C | T | KRAS | Missense | p.G12D | NM_033360 | 93 | 1347 | 6.90 | 2.E-04 | 4.E-195 |
| P31.1 | chr17 | 7579349 | A | C | TP53 | Missense | p.F113C | NM_001126113 | 33 | 880 | 3.75 | 2.E-04 | 9.E-59 |
| P31.2 | chrX | 76938208 | A | G | ATRX | Missense | p.F847S | NM_000489 | 18 | 540 | 3.33 | ND | 4.E-30 |
| P32.1 | chr12 | 25398284 | C | T | KRAS | Missense | p.G12D | NM_033360 | 44 | 2586 | 1.70 | 2.E-04 | 1.E-64 |
| P32.1 | chr17 | 7578271 | T | C | TP53 | Missense | p.H154R | NM_001126113 | 31 | 2441 | 1.27 | 2.E-04 | 3.E-41 |
| P36.1 | chr12 | 25398284 | C | T | KRAS | Missense | p.G12D | NM_033360 | 18 | 2384 | 0.76 | 2.E-04 | 5.E-19 |
| P36.1 | chr17 | 7578190 | T | C | TP53 | Missense | p.Y220C | NM_001126113 | 32 | 2724 | 1.17 | 2.E-04 | 2.E-41 |
| P36.2 | chr12 | 25398284 | C | T | KRAS | Missense | p.G12D | NM_033360 | 24 | 2378 | 1.01 | 2.E-04 | 5.E-29 |
| P36.3 | chr12 | 25398284 | C | T | KRAS | Missense | p.G12D | NM_033360 | 18 | 1646 | 1.09 | 2.E-04 | 9.E-22 |
| P36.3 | chr17 | 7578190 | T | C | TP53 | Missense | p.Y220C | NM_001126113 | 33 | 1962 | 1.68 | 2.E-04 | 1.E-47 |
| P36.4 | chr12 | 25398284 | C | T | KRAS | Missense | p.G12D | NM_033360 | 31 | 1804 | 1.72 | 2.E-04 | 6.E-45 |
| P36.4 | chr17 | 7578190 | T | C | TP53 | Missense | p.Y220C | NM_001126113 | 47 | 1919 | 2.45 | 2.E-04 | 2.E-76 |
| P36.2 | chr17 | 7578190 | T | C | TP53 | Missense | p.Y220C | NM_001126113 | 17 | 2485 | 0.68 | 2.E-04 | NS |
| P37.1 | chr9 | 21971101 | G | T | CDKN2A | Nonsense | p.C100X | NM_058195 | 22 | 1246 | 1.77 | 2.E-04 | 1.E-31 |
| P37.1 | chr12 | 25398284 | C | T | KRAS | Missense | p.G12D | NM_033360 | 90 | 1956 | 4.60 | 2.E-04 | 4.E-173 |
| P37.1 | chr17 | 7578457 | C | A | TP53 | Missense | p.R158L | NM_001126113 | 59 | 1425 | 4.14 | 2.E-04 | 3.E-110 |
| P37.2 | chr9 | 21971101 | G | T | CDKN2A | Nonsense | p.C100X | NM_058195 | 17 | 1472 | 1.15 | 2.E-04 | 5.E-21 |
| P37.2 | chr12 | 25398284 | C | T | KRAS | Missense | p.G12D | NM_033360 | 40 | 2275 | 1.76 | 2.E-04 | 3.E-59 |
| P37.2 | chr17 | 7578457 | C | A | TP53 | Missense | p.R158L | NM_001126113 | 27 | 1620 | 1.67 | 2.E-04 | 9.E-39 |
| P42.5 | chr7 | 100417264 | A | C | EPHB4 | Missense | p.F404L | NM_004444 | 71 | 2665 | 2.66 | 2.E-04 | 5.E-119 |
| P42.5 | chr11 | 108201015 | G | A | ATM | Missense | p.R2461H | NM_000051 | 20 | 1504 | 1.33 | ND | 5.E-26 |
| P42.5 | chr18 | 48591918 | C | T | SMAD4 | Missense | p.R361C | NM_005359 | 145 | 1413 | 10.26 | 2.E-04 | 0.E+00 |
| P42.5 | chr20 | 57415495 | G | A | GNAS | Missense | p.E112K | NM_016592 | 17 | 1433 | 1.19 | ND | 6.E-21 |
| P42.1 | chr17 | 7577144 | A | G | TP53 | Missense | p.L226P | NM_001126113 | 5 | 1377 | 0.36 | 3.E-04 | NS |
| P42.1 | chr18 | 48591918 | C | T | SMAD4 | Missense | p.R361C | NM_005359 | 7 | 1659 | 0.42 | 2.E-04 | NS |
| P42.1 | chr7 | 100417264 | A | C | EPHB4 | Missense | p.F404L | NM_004444 | 9 | 1842 | 0.49 | 2.E-04 | NS |
| P42.5 | chr17 | 7577144 | A | G | TP53 | Missense | p.L226P | NM_001126113 | 34 | 2027 | 1.68 | 2.E-04 | NS |
| P43.1 | chr1 | 27102188 | A | G | ARID1A | Missense | p.N1705S | NM_006015 | 43 | 1517 | 2.83 | ND | 4.E-72 |
| P43.1 | chr3 | 178927410 | A | G | PIK3CA | Missense | p.I391M | NM_006218 | 26 | 1619 | 1.61 | ND | 3.E-36 |
| P43.1 | chr11 | 108106443 | T | A | ATM | Missense | p.D126E | NM_000051 | 29 | 1171 | 2.48 | ND | 4.E-46 |
| P43.1 | chr11 | 108121733 | G | A | ATM | Missense | p.G514D | NM_000051 | 19 | 1620 | 1.17 | ND | 3.E-23 |
| P43.1 | chr11 | 108143456 | C | G | ATM | Missense | p.P1054R | NM_000051 | 22 | 1649 | 1.33 | ND | 2.E-28 |
| P43.1 | chr11 | 108159732 | C | T | ATM | Missense | p.H1380Y | NM_000051 | 23 | 1648 | 1.40 | ND | 2.E-30 |
| P43.1 | chr12 | 25398284 | C | T | KRAS | Missense | p.G12D | NM_033360 | 24 | 1566 | 1.53 | 2.E-04 | 9.E-33 |
| P43.1 | chr17 | 7577505 | T | A | TP53 | Missense | p.D259V | NM_001126113 | 16 | 1105 | 1.45 | 2.E-04 | 1.E-20 |
| P43.4 | chr2 | 29416520 | A | G | ALK | Missense | p.M1478T | NM_004304 | 42 | 3499 | 1.20 | ND | 3.E-55 |
| P43.5 | chr12 | 25398284 | C | T | KRAS | Missense | p.G12D | NM_033360 | 37 | 2279 | 1.62 | 2.E-04 | 3.E-53 |
| P43.1 | chr9 | 21971111 | G | A | CDKN2A | Missense | p.A97V | NM_058195 | 5 | 830 | 0.60 | 2.E-04 | NS |
| P43.4 | chr17 | 7577505 | T | A | TP53 | Missense | p.D259V | NM_001126113 | 4 | 2404 | 0.17 | 8.E-04 | NS |
| P43.5 | chr9 | 21971111 | G | A | CDKN2A | Missense | p.A97V | NM_058195 | 9 | 1566 | 0.57 | 3.E-04 | NS |
| P43.5 | chr17 | 7577505 | T | A | TP53 | Missense | p.D259V | NM_001126113 | 13 | 1675 | 0.78 | 2.E-04 | NS |
| P46.1 | chr12 | 25398284 | C | T | KRAS | Missense | p.G12D | NM_033360 | 10 | 1394 | 0.72 | 2.E-04 | NS |
| P46.1 | chr17 | 7578431 | G | A | TP53 | Missense | p.Q167X | NM_001126113 | 8 | 1284 | 0.62 | 2.E-04 | NS |

AA, amino acid; ND, not detected; NS, not significant; NT, nucleotide.

**Supplementary Table S7. Correlation of ctDNA and CA19-9 levels with therapy response evaluation.**

| **Patient ID** | **ctDNA** | | **CA 19-9** | |
| --- | --- | --- | --- | --- |
|  | **CR/PR** | **PD** | **CR/PR** | **PD** |
| **P2** | NR | + | NR | + |
| **P5** | + | + | + | - |
| **P7** | + | + | - | + |
| **P23** | + | NR | + | NR |
| **P31** | + | NR | NR | NR |
| **P36** | NR | + | NR | - |
| **P42** | NR | + | NR | + |
| **P43** | + | + | - | + |
| **positive / total** | 5/5 | 6/6 | 2/4 | 4/6 |
| NR, not related |  |  |  |  |
